# Protocol for project FIERCE: A randomized controlled trial to evaluate a positive emotion-focused meditation intervention for physicians with elevated stress

**DOI:** 10.1371/journal.pone.0357906

**Published:** 2026-09-09

**Authors:** J. Richard T. Korecki, Diana Winston, Elizabeth Ko, Sun Yoo, Joshua Khalili, Julienne E. Bower

**Affiliations:** 1 Department of Psychology, University of California, Los Angeles, California, United States of America; 2 UCLA Mindful, UCLA, Los Angeles, California, United States of America; 3 Department of Medicine, David Geffen School of Medicine at the University of California, Los Angeles, California, United States of America; 4 UCLA Integrative Medicine Collaborative, Los Angeles, California, United States of America; 5 UCLA Jonsson Comprehensive Cancer Center, Los Angeles, California, United States of America; 6 Department of Psychiatry and Biobehavioral Sciences, David Geffen School of Medicine, UCLA, Los Angeles, California, United States of America; 7 Cousins Center for Psychoneuroimmunology, Semel Institute for Neuroscience and Human Behavior, Los Angeles, California, United States of America; National Trauma Center, NEPAL

## Abstract

**Introduction:**

Nearly 80% of healthcare providers experience adverse psychological symptoms (e.g., depression, burnout, sleep disturbance) stemming from workplace stressors. Elevated levels of stress have been associated with unfavorable occupational, patient, and provider-related outcomes, imposing a heavy burden on a strained system. Given the impact of stress on both employee and patient health, effective interventions are urgently needed to reduce distress and promote well-being among healthcare professionals. Mindfulness-based interventions show promise for addressing these challenges. We developed a six-week, remotely delivered mindfulness intervention, the Building Emotional Strength Training (BEST) program, based on the Buddhist Four Immeasurables practice to cultivate the distinct emotional qualities of loving-kindness, compassion, joy, and equanimity. The present study aims to evaluate the feasibility and efficacy of a Four Immeasurables-based mindfulness intervention on perceived stress (primary outcome), burnout, depressive symptoms, and inflammatory biomarkers, while enhancing psychological well-being and sleep quality (secondary outcomes) in physicians. We will also investigate potential mediators of intervention effects, including compassion, positive affect, equanimity, and mindfulness.

**Method:**

We will enroll 90 full-time physicians in a remote, two-arm randomized controlled trial with 1:1 allocation to either the meditation intervention or waitlist control. Participants will complete self-report questionnaires and provide blood samples at baseline, mid-course, and post-intervention to assess outcomes and mediators.

**Discussion:**

The project aims to advance the study of mindfulness-based interventions that reduce distress and promote well-being through practices that cultivate prosocial and altruistic feelings toward oneself and others. While mindfulness interventions have gained considerable interest, none have specifically drawn from the Four Immeasurables practice to target loving-kindness, compassion, joy, and equanimity. This novel investigation could expand our understanding of practices that foster kindness and compassion to reduce distress in an at-risk population.

**Trial registration:**

ClinicalTrials.gov NCT07283744, registered on 2025/10/14. The Open Science Framework, registered on 2026/06/26.

## Introduction

Healthcare providers face a large number of stressful situations daily, which may increase their risk of developing adverse mental and physical health conditions [1–4]. Downstream consequences of working in high-stress healthcare occupations include elevated levels of stress, depressive symptoms, and burnout. It is estimated that frontline healthcare workers, including physicians, experience higher rates than the general population of stress, depression [3,5], and burnout [6–9]. These conditions have been associated with unfavorable occupational [10–22], provider [23–28], and organizational outcomes [10,17,29–31]. Among healthcare workers, physicians face a distinct constellation of stressors, including high decision-making and diagnostic burden [32], long and irregular hours [33], and a lack of organizational support [32,33], that may not be fully addressed by interventions developed for the broader workforce.

In addition to the occupation-specific outcomes associated with increased levels of distress, growing evidence suggests that stress exposure can influence physical and mental health-related outcomes (e.g., cardiovascular disease, diabetes, depression, etc.) by stimulating pro-inflammatory biology [34–36]. In fact, frontline healthcare workers experience high rates of physical complaints [1,2,37–41] that may be due to elevated inflammatory activity. Additionally, elevated inflammation may adversely affect cognitive, emotional, and behavioral outcomes [42–44], further impairing healthcare providers’ effectiveness.

Although much of the literature identifies organizational and structural factors, such as workload, administrative burden, and scheduling, as central drivers of physician stress and burnout [33], individual-level interventions may offer complementary tools that help physicians manage the distress these conditions produce [45]. To date, several attempts have been made to address psychological distress among healthcare providers using individual-focused behavioral interventions, with mixed results [45–48]. Given the encouraging effects of mindfulness-based interventions (MBIs) on various outcomes in diverse populations [49,50], it is unsurprising that these interventions have been used to address the needs of healthcare workers. Recent reviews have investigated the effects of several MBIs on various outcomes in healthcare provider populations with mixed results. Meta-analytic data suggest that while Mindfulness-Based Stress Reduction (MBSR) has demonstrated beneficial effects on reducing experiences of anxiety, depression, and perceived stress, the intervention was not effective at reducing symptoms of burnout [51]. These results may suggest that the specific type of mindfulness practice may matter for addressing the distress physicians experience. Interventions drawing on diverse contemplative practices (e.g., loving-kindness and compassion-based) remain largely unexamined in this population. Because physicians face substantial time and scheduling constraints, remote delivery may also improve the feasibility and reach of such interventions [52].

One of the ultimate goals of many MBIs, including MBSR, is to familiarize participants with what it means to be aware (i.e., mindful). Thus, the practices included in the interventions are designed to strengthen one’s ability to recognize when their awareness has drifted from the present moment and how to bring their focus back to the here and now [53]. An intervention aimed at enhancing positive psychological states may be more effective at reducing distress in this population. One such meditation-based intervention is the Four Immeasurables, also known as the Brahmaviharas in Pali.

The Four Immeasurables were described in the Fifth Century in one of the primary treatises on Buddhist practice, the Visuddhimagga [54], and include the distinct emotional qualities of loving-kindness, compassion, sympathetic joy, and equanimity [55]. These emotional states are gradually cultivated through specific practices that often include visualizing oneself and others and directing the feeling state of the particular component (e.g., loving-kindness) to one’s target by mentally reciting phrases such as “may you live with ease.”

Although the Four Immeasurables have been practiced in traditional Buddhist settings for thousands of years, the entire four-factor program has not been investigated in a randomized controlled trial (RCT). Despite a shortage of trials investigating the complete Four Immeasurable practice, there is evidence from RCTs evaluating interventions that include singular components of the Four Immeasurables, as well as from cross-sectional studies demonstrating the benefits of experiencing kindness, compassion, joy, and equanimity. These investigations suggest that these practices and emotional states can beneficially impact psychological, social, and biological outcomes [56–64]. Additionally, these four psychological constructs may be particularly beneficial in lessening the common occupation-related symptoms experienced by healthcare providers, namely perceived stress, depression, and burnout [60,65,66]. Furthermore, the favorable social influences that the Four Immeasurables appear to foster [60,67,68] may suggest that these practices would significantly benefit individuals in caregiving occupations, thereby improving patient outcomes and enhancing provider well-being.

### Objectives and aims

Our team has developed a manualized mindfulness intervention, the Building Emotional Strength Training (BEST) program, based on the traditional Four Immeasurables practice to reduce stress among physicians. In this article, we describe the protocol for a single-site, two-arm, parallel-group RCT evaluating the feasibility and efficacy of the BEST intervention, delivered remotely, compared with a waitlist control (WLC) for perceived stress among physicians. Ninety physicians with elevated stress will be randomized to BEST or a WLC condition. Aim 1 is to assess the feasibility of delivering the BEST intervention in a workplace setting to full-time healthcare workers. Aim 2 is to determine the effects of BEST vs. WLC on perceptions of stress (primary outcome). Aim 3 is to evaluate the effects of BEST vs. WLC on negative (depressive symptoms and burnout) and positive psychosocial symptoms (psychological well-being and sleep quality; secondary outcomes) experienced by healthcare workers. Aim 4 is to determine the effects of BEST vs. WLC on inflammatory processes associated with stress and relevant to long-term health. Lastly, an exploratory aim will examine theoretically based mechanisms (compassion for oneself and others, positive emotions, equanimity, and mindfulness) of the effect of the BEST intervention on the primary outcome, perceived stress.

## Methods

**Fig 1:**
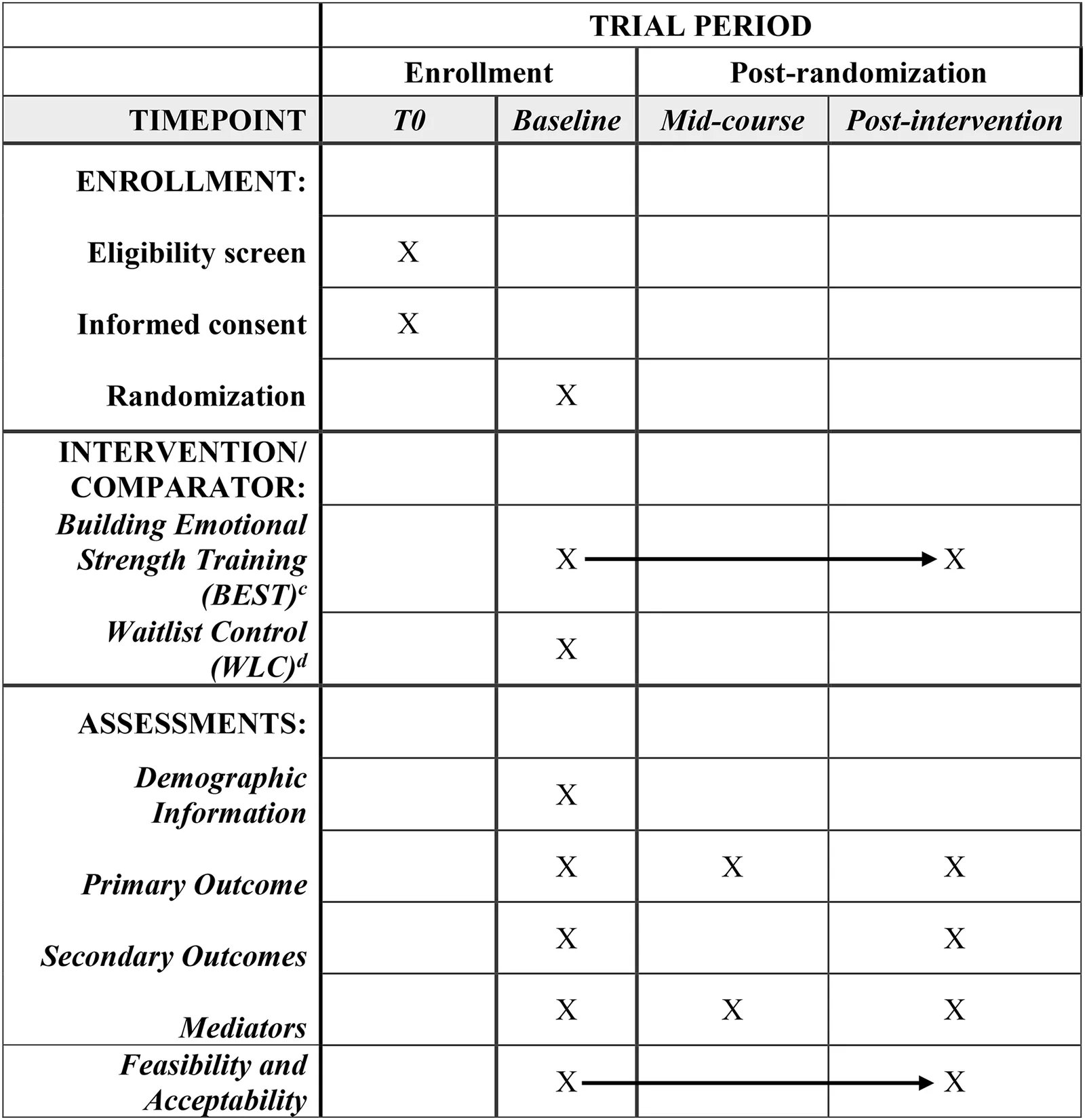
Participant timeline: Schedule of enrollment, interventions, and assessments.

**Fig 2:**
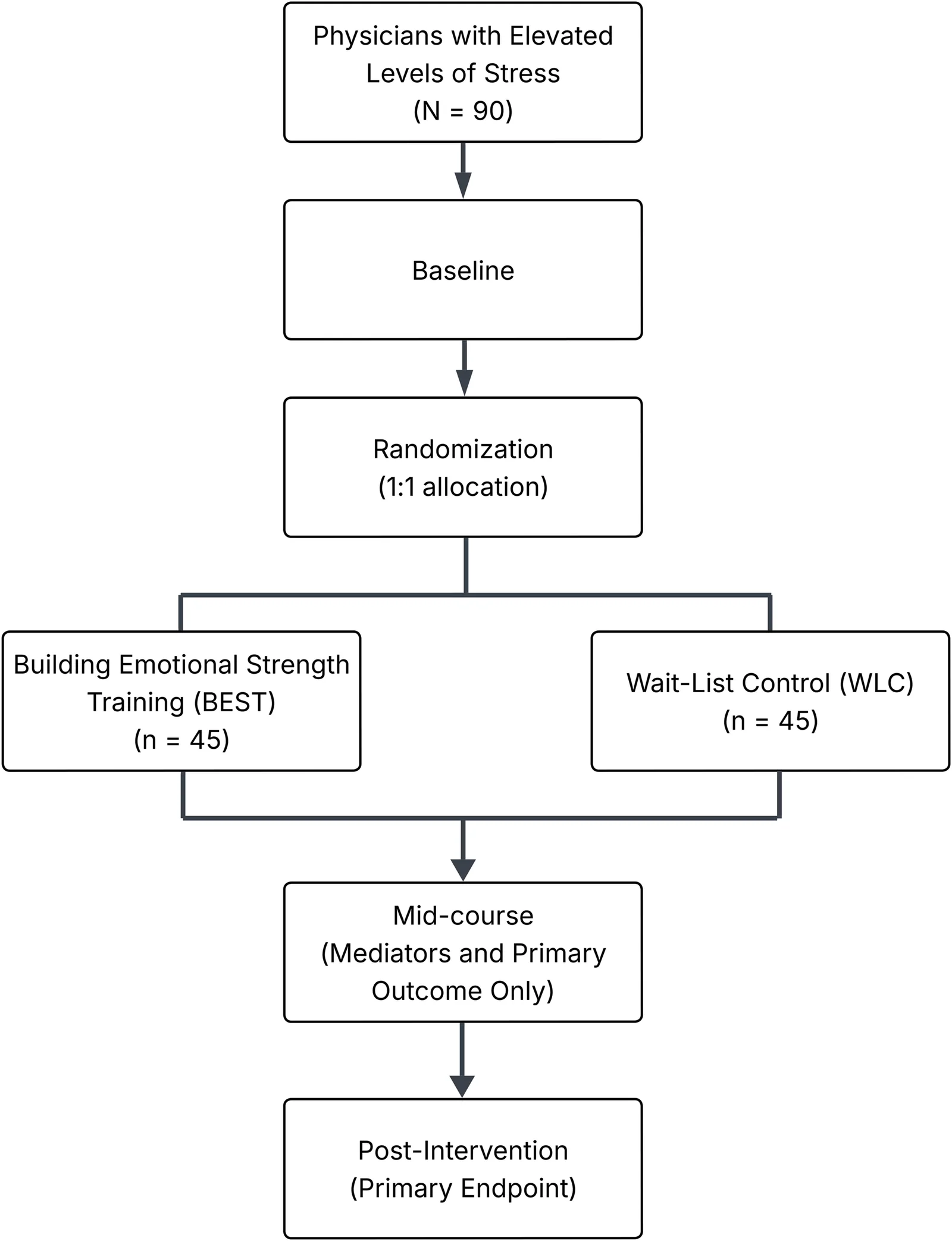
Participant Flow Diagram.

The study flow and procedures are detailed in Figs 1 and 2. The trial was registered on ClinicalTrials.gov (NCT07283744) before recruitment, specifying the study design, arms, eligibility criteria, and primary and secondary outcomes. The detailed analysis plan was subsequently registered on the Open Science Framework (OSF; osf.io/npcax) before any access to outcome data. This trial has received Institutional Review Board (IRB) approval from the University of California, Los Angeles IRB (IRB-25–1518) and will be conducted and reported in accordance with CONSORT guidelines [69]. Any adverse or serious events will be reported to the principal investigators and the institutional review board within 24 hours.

Using a 1:1 allocation, we will randomize 90 participants to the BEST intervention (n = 45) or WLC (n = 45). Primary, secondary, and exploratory outcomes will be assessed at baseline, mid-course, and post-intervention (after week 6 of the six-week intervention).

### Setting

This study is conducted through the University of California, Los Angeles (UCLA), which serves as the IRB of record. Because all assessments and intervention classes are administered remotely, participants are recruited from across different healthcare systems and are not required to live in the greater Los Angeles area. Regardless of participant location, all data and study materials (e.g., biological specimens) will be securely stored at UCLA.

### Trial status and timeline

Participant recruitment began on December 02, 2025, and is expected to conclude by October 2026. Data collection will continue until December 2026. Data analysis will be conducted once data collection is complete, with results expected in early 2027.

### Participants

Eligibility criteria include: 1) currently practicing physician (M.D. or D.O.), 2) at least 18 years of age, 3) willing and able to attend the six-week intervention, 4) fluent in English, 5) access to the internet and email, and 6) score of four or greater on the four-item Perceived Stress Scale [PSS-4; 70]. Perceived stress will be assessed using the PSS-4 during the initial phone screening. Exclusion criteria include: 1) having a current and consistent meditation practice (i.e., engaging in a formal meditation practice at least three days per week for five minutes each day), 2) previous participation in a Four Immeasurable course (e.g., attending a Brahmaviharas meditation retreat), 3) regular tobacco use (given its impact on immune function; [70]). Eligibility is intentionally broad to enhance generalizability to the physician population. We do not exclude participants on the basis of medical or psychiatric conditions or medication use. We do, however, assess these characteristics at baseline. Participants report current medication use (including dosage, frequency, and reason for use) and provide information on any diagnosed psychiatric conditions, use of mental health services, and the frequency of such use. These data are collected to characterize the sample and are available for use in sensitivity or exploratory analyses.

### Recruitment of healthcare providers

Relationships with senior staff members of several departments within the UCLA Health System will be leveraged to identify and recruit potential participants. We have enlisted the assistance of several healthcare professionals at UCLA who focus on the health and well-being of UCLA Health employees. These individuals were consulted on the design of the intervention and format of the study. Additionally, they offered to leverage their networks to help advertise the project and facilitate participation (e.g., adjusting work schedules so that clinicians have dedicated time built into their schedules to participate in the trial). Further, other recruitment sources will be explored, including 1) announcements at staff meetings, 2) posting study flyers around the UCLA Health campus, 3) attendance at provider wellness events, and 4) advertisements in provider-focused newsletters.

### Procedures and study flow

*Eligibility Screening, Informed Consent, and Baseline Assessment (T1).* Interested individuals will contact the study team via email or phone. The principal investigator will then call potential participants to confirm eligibility criteria. Participants will be recruited in cohorts of 20–30 physicians, who will be randomly assigned to either the BEST group or the WLC, allowing for our desired mindfulness group size of 10–15 participants. Screening will continue until we have an adequate number of eligible, interested, and available participants for the next cohort.

All eligible participants will then review and verbally acknowledge the IRB-approved consent form. After obtaining consent and enrollment, participants will be automatically emailed a link to complete the study questionnaires through Qualtrics. They will also receive an at-home blood collection kit in the mail to provide a blood sample for immune evaluation. Participants will be advised to complete the blood collection in the morning to control for diurnal variations in immune parameters. Based on pilot completion data, the questionnaire battery takes approximately 20–30 minutes, and blood specimen collection requires approximately 10 minutes. Baseline assessments will be conducted during the 2 weeks preceding the first class of the intervention. The principal investigator conducts eligibility screening and consent, and the distribution of assessment links is automated through Qualtrics.

*Randomization.* A block randomization sequence was generated prior to enrollment using the ‘blockrand’ function in R [71], allocating participants 1:1 to the two study conditions. Because the sequence was fixed in advance, individual assignments could not be altered during the trial. The principal investigator conducts eligibility screening, informed consent, baseline assessment, and, upon completion of baseline, notifies participants of their assigned condition. We acknowledge that, because these roles are held by a single individual who also has access to the master allocation list, allocation was not concealed through personnel separation. Instead, allocation concealment relied on the pre-generation of the randomization sequence and adherence to the pre-specified procedure of not consulting assignments until after baseline completion. The use of predefined, largely objective eligibility criteria (degree, age, language, meditation practice, PSS-4 cutoff) further limits the potential for assignment foreknowledge to influence enrollment. This limitation is noted in the Discussion.

*Blinding.* Given the nature of a mindfulness intervention [72], this is an open-label trial. Participants and intervention instructors are necessarily aware of group assignment, and the principal investigator, who also conducts enrollment, assessment, and analysis, is unblinded to allocation. Allocation was not concealed through personnel separation; as described above (see *Randomization*), the pre-generated sequence and predefined eligibility criteria instead limit the potential for foreknowledge to bias enrollment. To reduce the risk of bias arising from the open-label design, we (1) include an objective, inflammation-based outcome not subject to self-report bias; (2) distribute assessment links automatically through Qualtrics rather than by the principal investigator, separating outcome-data collection from personnel aware of allocation; (3) restrict access to the raw Qualtrics outcome dataset to the principal investigator; and (4) commit that the principal investigator will not access outcome data for analysis until data collection is complete, with all primary and secondary outcomes and the analysis plan preregistered before analysis (ClinicalTrials.gov NCT07283744; OSF osf.io/npcax), together constraining analytic flexibility despite unblinded analysis.

*Mid-Course Assessment (T2).* At the halfway point of the intervention (week 3), all participants will be emailed a link to complete self-report questionnaires assessing only the primary outcome and the mediator variables. The reduced midpoint battery takes approximately 5–10 minutes to complete.

*Post-Intervention Assessment (T3).* All participants will be emailed the post-intervention assessment link and mailed a second blood collection kit to complete both tasks within 2 weeks following the conclusion of the study intervention. The questionnaire will include all study variables. Assessment burden at post-intervention is equivalent to baseline (approximately 20–30 minutes for questionnaires and 10 minutes for blood collection).

*Procedure for Blood Collection, Processing, and Storage.* The Tasso T-20 device [73] will be used to collect, store, and transport capillary blood for testing at the Social Genomics Core Laboratory. The Tasso is designed for use in non-clinical settings with adult populations. After collection, samples will be stored at −20 °C and analyzed in a single batch once data collection is complete.

### Intervention conditions

*General Information.* The experimental mindfulness intervention is a manualized six-session (60 minutes each) protocol designed for in-person or remote delivery by trained mindfulness instructors. The intervention will be group-based and delivered remotely via Zoom. Based on input from our community advisors, the group sessions will be conducted during the workday (around lunchtime).

*Building Emotional Strength Training (BEST).* Diana Winston, Director of UCLA Mindful, developed the intervention drawing on her substantial experience working with meditation practitioners and her expertise in designing group-based mindfulness courses for diverse audiences [74]. Notably, the research team consulted with several physicians to determine the most suitable format, including class length, scheduling, and mode of delivery, to ensure the intervention was feasible and appropriate for our target population. These consultations informed the decision to deliver BEST remotely via videoconference, in shortened sessions scheduled at times that accommodate physicians’ clinical demands, to design a program that could fit within the busy schedule of a full-time physician.

As reviewed above, the traditional Four Immeasurables practice includes exercises designed to cultivate the distinct emotional states of loving-kindness, compassion, sympathetic joy, and equanimity. While the core practices are not altered, BEST incorporates light contextual adaptations for physicians, including content, examples, and informal practices framed in the language of clinical work (e.g., attending to a patient’s breath through a stethoscope as a cue for mindful awareness). The goal is to provide tools that can be deployed as needed in daily activities, especially during clinical duties. The BEST curriculum is a six-week program delivered in six weekly, one-hour, group-based videoconference sessions, each focused on a different component of the Four Immeasurables practice. Participants who attend at least three of the six BEST sessions will be considered to have completed the intervention. The practices introduced across BEST—mindfulness, loving-kindness, compassion, equanimity, and joy—each have independent empirical support for beneficial effects [50,60,75]. While the full program is designed to be delivered in its entirety and its components may confer additive benefit, the individual-level evidence indicates that even partial attendance exposes participants to practices with demonstrated value. Attending at least three sessions therefore represents a meaningful level of exposure rather than a negligible dose, supporting three of six as an adherence threshold. This threshold, consistent with prior work [76–78], is pre-specified to characterize intervention completion and to define the per-protocol sample in sensitivity analyses; it does not imply that partial attendance confers the full benefit of the complete program.

During each session, participants receive structured training and instruction in meditation exercises, including theoretical background, formal guidance on the day’s topic, and question-and-answer sessions to support learning. Additionally, participants will be guided through each meditation exercise introduced during class. Furthermore, each class will include dyadic discussions, allowing participants to interact directly with one another. The sessions will introduce the following topics and associated practices: 1) introduction to mindfulness, 2) mindfulness and self-compassion, 3) self-directed kindness, 4) compassion, 5) equanimity, and 6) joy. The BEST intervention also includes home practice, in which participants are encouraged to practice the meditation technique introduced in class at home, starting with five minutes and gradually increasing to 20 minutes per day throughout the six weeks of the intervention. All sessions will be delivered by the intervention developer, who has nearly 30 years of experience teaching meditation.

*Waitlist Control (WLC).* The WLC will control for naturally occurring changes in the outcomes and mediators over the assessment period. Participants in this group will not engage in any study-related activities outside of the remote assessments (baseline, mid-course, and post-intervention). Participants randomized to this condition will be offered the BEST intervention after all data collection is completed.

### Measures

Reliable, valid, and commonly used self-report questionnaires will be administered at baseline (T1), mid-course (T2), and post-intervention (T3) to assess participant characteristics and outcomes. All measures have been validated in adult populations and have been shown to be sensitive to the effects of psychosocial interventions. Questionnaires will be administered online using a Qualtrics link emailed to each participant. The complete list of measures is presented in Table 1.

**Table 1:** Assessment Schedule.

	T1	T2	T3
**Background characteristics**			
Demographic characteristics	X		
Employment	X		
Health history, Use of mental health services	X		X
Height and weight measurements	X		
Health behaviors/meds	X		X
Calendar Allocation and Continuing Medical Education Training			X
**Outcomes**			
Perceived stress (Perceived Stress Scale [PSS])	X	X	X
Burnout (Maslach Burnout Inventory for Medical Personnel [MBI-M])	X		X
Psychological well-being (Mental Health Continuum Short Form [MHC-SF])	X		X
Depressive symptoms (Center for Epidemiologic Studies – Depression [CES-D])	X		X
Sleep quality (Insomnia Severity Index [ISI])	X		X
Circulating inflammatory markers	X		X
Pro-inflammatory gene expression	X		X
**Mediators**			
Self-compassion (Self-Compassion Scale-Short Form [SCS-SF])	X	X	X
Compassion for others (Compassion Scale [CS])	X	X	X
Positive and negative emotions (modified Differential Emotions Scale [mDES])	X	X	X
Equanimity (Equanimity Scale [ES])	X	X	X
Mindfulness (Five Factor Mindfulness Questionnaire [FFMQ])	X	X	X
**Feasibility and Acceptability**			
Session attendance*	X	X	X
Retention*			X
Acceptability (Treatment Satisfaction Questionnaire [TSQ])			X
BEST Course Evaluation Form (Optional)			X

*Note. Attendance is recorded at each session; retention is derived from study records.

#### Feasibility and acceptability.

Feasibility will be evaluated based on participant recruitment, retention, and adherence. We will track enrollment relative to our recruitment target, retention through the post-intervention assessment, and attendance at the intervention group sessions. Feasibility will be assessed against the following pre-specified benchmarks: recruitment of 90 participants within the recruitment period; retention of  ≥ 80% of randomized participants through the post-intervention assessment (T3); and intervention adherence, defined as  ≥ 80% of intervention participants attending at least three of the six sessions (the pre-specified completion threshold).

Acceptability will be evaluated using two complementary instruments. First, all participants randomized to the intervention will complete an abbreviated and adapted version of the Treatment Satisfaction Questionnaire (TSQ) [79] at the post-intervention assessment (T3). The three-item TSQ determines the extent to which the participant views the intervention as helpful and beneficial [79]. Participants respond to three statements on an eight-point Likert scale ranging from 0 (not at all) to 7 (extremely), yielding a possible range of 0–21, with higher scores indicating greater satisfaction. Acceptability will be considered adequate if the mean TSQ score is ≥ 12.

Second, intervention participants are invited to complete an optional post-intervention feedback form designed to characterize acceptability across distinct dimensions and to identify which specific features of the intervention participants find more or less acceptable. The form includes five items rated on a five-point scale (1 = poor, 5 = excellent) that assess overall course quality, the relevance of the practices to daily life, clarity of instruction and facilitation, pacing and structure, and the likelihood of recommending the course. Rating these dimensions separately allows acceptability attributable to the content, the instruction, and the course structure to be distinguished rather than captured as a single global score. The form also includes open-ended questions that probe the acceptability of the intervention’s distinctive multi-practice content, including which of the practices (mindfulness, loving-kindness, compassion, equanimity, and joy) participants found most valuable, whether the number of practices felt manageable, and whether participants would have preferred a course focused solely on mindfulness, as well as perceived strengths, suggestions for improvement, and practices participants intend to maintain. Because the feedback form is optional and administered only to intervention-arm participants, these responses are considered exploratory and may not be representative of all participants. Responses are intended to contextualize and enrich the quantitative acceptability data rather than to provide confirmatory estimates.

#### Demographics and occupation-related variables.

Demographics, including age, sex, race/ethnicity, income, educational status, relationship status, and employment status, will be collected at baseline. Occupation-related variables, including provider role (hospitalist, primary care, specialist, or surgeon) and the shift typically worked (day, evening, or night), will be collected at baseline.

#### Primary outcome: Stress.

Stress will be assessed using the ten-item Perceived Stress Scale (PSS) [80]. The PSS will be administered at baseline (T1), mid-course (T2), and post-intervention (T3). The PSS evaluates how often individuals appraised situations in their lives as stressful during the past month. Respondents respond on a five-point Likert scale from 0 (never) to 4 (very often). All scores are summed to provide a total stress score. Scores range from 0 to 40, with higher scores indicating greater perceived stress. The PSS has been widely used with healthcare provider populations [81,82] and has been shown to be responsive to MBIs [83].

#### Secondary outcomes.

Secondary outcomes include depressive symptoms, burnout, psychological well-being, sleep quality, and markers of inflammation. They will be assessed at baseline (T1) and at the post-intervention time point (T3).

*Depressive symptoms*. Depressive symptoms will be based on scores on the 20-item Center for Epidemiologic Studies-Depression Scale (CES-D), a commonly used depression measure [84]. The CES-D is a reliable, validated, and widely used measure that includes central components of depressive symptomatology [84]. Participants respond to twenty statements that assess how often the individual felt or behaved during the past week on a four-point Likert scale from 0 (rarely or none of the time [less than 1 day]) to 3 (most or all of the time [5–7 days]). All 20 items will be summed to provide a total score. The possible range of scores is 0–60, with higher scores indicating a greater frequency of depressive symptoms. The CES-D has frequently been used to assess depressive symptoms in healthcare workers [29,85] and has been shown to be responsive to mindfulness interventions [86].

*Burnout.* Burnout will be measured via the 22-item Maslach Burnout Inventory for Medical Personnel (MBI-M) [87]. Items assess the frequency of personal attitudes and feelings within the past month [87]. The MBI-M measures three components of burnout: emotional exhaustion, depersonalization, and reduced personal accomplishment. Emotional exhaustion is assessed with nine items and refers to feeling emotionally overextended and exhausted by one’s work. Depersonalization is assessed with five items and refers to an impersonal response toward the recipients of one’s services. Personal accomplishment is assessed with eight items and reflects feelings of competence and achievement in work-related pursuits. All items are scored on a 7-point Likert scale from 0 (never) to 6 (every day). Higher scores on the emotional exhaustion and depersonalization subscales correspond to a greater experience of burnout. The personal accomplishment subscale is positively worded; therefore, a one-item increase corresponds to a greater experience of work-related efficacy. Each subscale will be summed to provide total scores for emotional exhaustion, depersonalization, and personal accomplishment. The MBI-M has been validated in several samples of healthcare workers [88,89] and has been shown to be sensitive to psychosocial intervention effects [90].

*Psychological well-being.* Well-being will be measured using the 14-item Mental Health Continuum Short Form (MHC-SF) [91,92]. The MHC-SF is a 14-item scale that measures three dimensions of psychological well-being: hedonic, eudaimonic-social, and eudaimonic–psychological. Hedonic well-being is measured with three items and refers to pleasurable states characterized by the presence of positive and absence of negative affect [91]. Eudaimonic social well-being is assessed via five items and refers to flourishing within a social context characterized by social coherence and actualization [93]. Eudaimonic psychological well-being is assessed with six items and refers to a sense of living a life of purpose, characterized by feelings of personal growth and self-actualization [91]. Respondents rate the 14 items to assess how often the statements were true in the past month, using a 6-point Likert scale from 0 (never) to 5 (every day). All scores are summed to provide a total well-being score. The possible range of scores is 0–70, with higher scores indicating greater subjective well-being. To our knowledge, the MHC-SF has not been used in a dedicated sample of healthcare providers. However, it has been shown to be reliable and valid in various adult populations [92,94]. Additionally, the MHC-SF has been shown to be sensitive to the effects of mindfulness interventions [95].

*Sleep quality.* Sleep quality will be assessed using the seven-item Insomnia Severity Index (ISI) [96]. The ISI is a reliable and valid self-report measure that evaluates the severity and impact of insomnia over the past two weeks. Respondents rate each of the seven items on a five-point Likert scale (0 = not at all, 4 = very much) to assess how they relate to each statement over the past two weeks. Scores are summed to obtain a total score. The possible range of scores on the ISI is 0–28, with higher scores indicating more severe insomnia. Additionally, scores of 15 or higher are indicative of clinically significant insomnia. The ISI has been validated for use with healthcare professionals [97] and shown to be responsive to mindfulness interventions [98].

*Inflammation.* We will utilize an integrated assessment of inflammation that encompasses systemic levels of inflammatory markers, inflammatory gene expression, and promoter-based bioinformatics analyses of specific inflammation-related transcription factors. Each measurement level provides unique and complementary information, offering a comprehensive view of inflammatory activity.

*Circulating inflammatory markers.* Assays will focus on pro-inflammatory cytokines and markers of inflammation that are responsive to mind-body interventions [99,100]. Meso Scale Discovery multiplex immunoassays will be used to assess five key pro- and anti-inflammatory cytokines (interleukin [IL]-6, IL-8, IL-10, tumor necrosis factor [TNF]-α, and interferon [IFN]-γ), and R&D Systems ELISA will assess C-reactive protein (CRP). Previous mindfulness studies have demonstrated decreases in CRP [101,102] and IL-6 [103,104].

*Molecular analysis of pro-inflammatory gene expression and signaling.* Primary analyses will focus on the expression of 19 pro-inflammatory genes shown to be upregulated in the context of chronic stress [105]. This set of genes, the pro-inflammatory component of the Conserved Transcriptional Response to Adversity (CTRA), has been shown to be responsive to mindfulness interventions [103,106,107]. Additionally, we will examine effects on 28 Type I interferon response genes and three antibody synthesis genes, also components of the CTRA.

Further, we will assess pro-inflammatory gene expression at the molecular level using a novel bioinformatics approach. This approach uses genome-wide transcriptional profiling to identify genes differentially expressed in leukocytes from participants randomized to the BEST group compared with those randomized to the WLC. Then, the approach uses the Transcription Element Listening System (TELiS) transcription factor search engine to identify the activation of specific transcription control pathways, including nuclear factor kappa-light-chain-enhancer of activated B cells (NF-κB), activator protein-1 (AP-1), cAMP-responsive element-binding protein (CREB), and glucocorticoid receptor (GR) signaling, which are hypothesized to underlie increased inflammatory activity. To identify the cellular sources of differentially expressed genes, we will carry out Transcript Origin Analysis [108]. Genomic assays for inflammatory markers will be conducted at the UCLA Social Genomics Core Laboratory under the supervision of Steve Cole, PhD. Biological samples are labeled with participant study codes rather than with treatment assignment codes. Therefore, laboratory personnel processing the inflammatory biomarker and gene-expression samples are blinded to treatment allocation.

*Exploratory outcome: mediators of intervention effects on stress.* Selected mediators include compassion for self and others, positive emotions, and equanimity/mindfulness. They will be assessed at baseline (T1), the study midpoint (T2), and post-intervention (T3).

*Compassion (self and others).* Self-compassion will be assessed using the 12-item Self-Compassion Scale-Short Form (SCS-SF) [109,110]. The SCS-SF assesses an individual’s global tendency to show kindness, concern, and sympathy for one’s own suffering [110]. The scale has six subscales with two items each: self-kindness (compassion for oneself during difficult moments), self-judgment (disapproval or intolerance of oneself), common humanity (appreciation of the universal nature of having flaws and inadequacies), isolation (experiencing failures alone), mindfulness (balance and poise during difficult moments), and over-identification (obsessing over negative feelings). Individuals respond to how often they generally behave in a stated manner on a five-point Likert scale from 1 (almost never) to 5 (almost always) in response to the 12 items. The six items from the self-judgment, isolation, and overidentification subscales are reverse-scored (i.e., 1 = 5, 2 = 4, etc.). The mean of each subscale is then calculated to compute a total mean (the average of the six subscale means). Scores range from 1–5, with higher scores indicating greater self-compassion. The SCS-SF has been used in the context of an intervention for healthcare workers and demonstrated sensitivity to changes following the intervention [111].

Compassion for others will be assessed using the 16-item Compassion Scale (CS) [112]. The CS comprises four subscales, each with four items, that determine how one generally reacts toward others. The four subscales include kindness (compassion and caring towards others during times of difficulty), common humanity (appreciation for the universal experience of pain), mindfulness (awareness of others and their experiences), and indifference (an unsympathetic demeanor towards others’ suffering). Individuals answer how often they generally behave in a stated manner on a five-point Likert scale from 1 (almost never) to 5 (almost always) in response to the 16 items. The four items on the indifference subscale are reverse-scored (i.e., 1 = 5, 2 = 4, etc.). The mean of each subscale is then calculated to compute a total mean (the average of the six subscale means). Scores range from 1–5, with higher scores indicating greater feelings of compassion for others. The CS has been used in healthcare worker populations [113]. To the best of our knowledge, the CS has not yet been used in an intervention setting.

*Positive and negative emotions.* Positive emotions will be assessed using the 20-item modified Differential Emotions Scale (mDES) [114]. The questionnaire includes ten items assessing positive emotions, such as contentment, amusement, and awe, and ten items assessing negative emotions, such as sadness, shame, anger, and hatred. The mDES attempts to capture the experience of both high- and low-arousal positive and negative emotions [114]. For each item, respondents indicate the extent to which they felt a specific emotion in the past 24 hours on a five-point Likert scale, ranging from 0 (not at all) to 4 (extremely). Each subscale (positive and negative) is independent of the other, so the mDES will yield a total score for both positive and negative emotions. Each subscale item is summed to provide total positive and negative emotion scores. Scores on each subscale range from 0 to 40, with higher scores on the positive subscale indicating a greater experience of positive emotions and higher scores on the negative subscale indicating a greater experience of negative emotions. The mDES has previously been used in healthcare populations [115] and has been shown to be influenced by mindfulness interventions [116,117].

*Equanimity.* Equanimity will be assessed using the 16-item Equanimity Scale (ES-16) [118]. The ES-16 assesses an individual’s tendency to respond in a balanced, calm, and even-tempered manner to all experiences (positive or negative). The scale comprises two subscales, each consisting of eight items: experiential acceptance (neutrality toward all internal experiences) and non-reactivity (an evenness of response toward all experiences). Individuals respond on a five-point Likert scale from 1 (strongly disagree) to 5 (strongly agree) to indicate how they view themselves. The eight items on the non-reactivity subscale are reverse-scored. All 16 items are summed to provide a total equanimity score. Scores range from 16–80, with higher scores indicating greater equanimity. The scale has not been used with healthcare professionals, but it has been shown to be sensitive in detecting changes following a mindfulness intervention [119].

*Mindfulness.* Mindfulness will be assessed using the 15-item Five-Factor Mindfulness Questionnaire (FFMQ-15) [120,121]. The FFMQ assesses five distinct facets of mindfulness using five subscales: observing, describing, acting with awareness, non-judging, and non-reactivity. Individuals respond on a five-point Likert scale, ranging from 1 (never or very rarely true) to 5 (very often or always true), to indicate how each statement relates to their general attitude. All items are summed and divided by 15 to provide an average mindfulness score. Scores range from 1–5, with higher scores indicating greater mindfulness. The FFMQ has been used with healthcare workers [122] and has been shown to be sensitive to the effects of mindfulness interventions in this population [123].

### Sample size

*Primary and secondary outcome analyses.* Typical effect sizes from randomized controlled trials of MBIs with passive control groups range from ds = 0.10–0.89, with trials in healthcare populations achieving an average effect size of 0.39 and digitally delivered interventions achieving an average effect size of 0.54 [124]. Recruitment capacity for the current trial was 90 participants, given financial and time constraints. Accordingly, we report the minimum detectable effect with 80% power for the primary outcome, perceived stress at the post-intervention assessment, comparing the mindfulness condition with the waitlist control (WLC). The minimum detectable effect was estimated using G*Power [125] for the primary analysis of covariance (ANCOVA; fixed effects, a two-level between-subjects factor, and one covariate), with the baseline value of the outcome entered as the covariate, α = 0.05, power = 0.80, and one numerator degree of freedom. Assuming a baseline–post correlation for PSS of r = 0.50 and the anticipated enrolled sample of 90 participants (45 per condition), of whom approximately 72 are expected to provide complete outcome data after accounting for ~19% attrition, based on a meta-analysis of overall and differential attrition across 114 randomized MBI trials in clinical and non-clinical populations [126], the study provides 80% power to detect a between-group effect of d = 0.58 (Cohen’s f = 0.34). This effect is comparable to the average effect of digitally delivered MBIs relative to passive control conditions (d = 0.54) [124], indicating that the trial is powered to detect effects of the magnitude typically observed for remotely delivered MBIs. In sensitivity analyses varying the baseline–post correlation from r = 0.40 to 0.60, the minimum detectable effect ranged from d = 0.53 to 0.61. The trial is powered for the primary comparison on perceived stress at the post-intervention endpoint. Analyses of secondary outcomes (burnout, depressive symptoms, inflammatory biomarkers, psychological well-being, and sleep quality) will provide estimates of intervention effect sizes but are not powered for definitive hypothesis testing.

*Power for exploratory mediation analyses*. Power for the exploratory parallel mediation model was estimated via Monte Carlo simulation using the application of Schoemann, Boulton, and Short [127]. The model specified group assignment (BEST vs. WLC) as the predictor; compassion, positive emotions, and equanimity/mindfulness at T2 as parallel mediators; and perceived stress (PSS) at T3 as the outcome, with baseline (T1) values of the outcome and each mediator included as covariates. Each specific indirect effect was estimated as the product of its *a* (group→mediator) and *b* (mediator→outcome) paths and tested using a Monte Carlo confidence interval. Intervention-to-mediator effects were derived from meta-analytic estimates comparing mindfulness-based programs with passive control conditions (Hedges’ g = 0.36, 0.44, and 0.22 for compassion, positive emotions, and mindfulness, respectively [75,124,128,129]). Since these are standardized mean differences for a two-group contrast, they were converted to point-biserial correlations for entry into the model (r = g/√(g² + 4), yielding 0.18, 0.22, and 0.11). Mediator–outcome correlations were drawn from prior longitudinal work (r = −0.40, −0.47, and −0.65, respectively [130–132]); the group–outcome correlation was set to −0.24 (corresponding to the anticipated *d* = 0.50 on the primary outcome), and inter-mediator correlations were set to 0.40 (range examined: 0.35–0.45). Based on the anticipated analyzable sample (N = 72; 90 enrolled, ~ 19% attrition), estimated power for each specific indirect effect was low (all < 0.30). Consistent with their preregistered designation, these mediation analyses are therefore exploratory and hypothesis-generating, and the resulting estimates are intended to inform effect-size assumptions for future, adequately powered mediation studies.

### Data analysis plan

*Descriptive analyses.* Descriptive statistics will be created to show the demographic and psychological characteristics of the sample. Baseline demographic and occupation-related characteristics will be summarized descriptively by condition (BEST and WLC) using means and standard deviations for continuous variables and frequencies and percentages for categorical variables. In addition to standard demographic characteristics, we will summarize sources of heterogeneity within the physician sample, including medical specialty, workload, and career stage, to characterize the sample’s composition across these dimensions. No inferential tests of baseline differences will be conducted. Consistent with our preregistered analysis plan, the primary outcome models adjust for the baseline value of the outcome, regardless of observed baseline balance. Additionally, we will examine the distributional properties of the study variables to assess normality. We will address issues of non-normality using applicable techniques, including robust standard errors and rescaling. It is common practice to log-transform inflammatory markers to normalize distributions.

*Feasibility and acceptability.* Analyses of feasibility and acceptability will be primarily descriptive and will be evaluated against the pre-specified benchmarks described above. Feasibility will be summarized as the number of participants recruited during the recruitment period, the proportion retained through the post-intervention assessment, and session attendance. Attendance will be reported as the number of sessions attended (including the full distribution across participants) and as the proportion of participants who meet the completion threshold of at least three of six sessions. Acceptability will be summarized using mean Treatment Satisfaction Questionnaire (TSQ) scores, evaluated against the pre-specified acceptability benchmark, together with the dimension-specific ratings from the optional feedback form (overall quality, relevance, clarity of instruction, and pacing/structure), reported separately to characterize distinct components of acceptability. The open-ended feedback form responses will be summarized qualitatively to identify specific features that participants found more or less acceptable. Where sample size permits, feasibility and acceptability will additionally be summarized across relevant subgroups (e.g., specialty) to examine heterogeneity. These subgroup summaries will be descriptive and exploratory.

*Primary and secondary outcomes.* All analyses will employ an intention-to-treat (ITT) principle. Thus, regardless of how many classes they attended or assessments they completed, all randomized participants will be included in analyses and analyzed based on their original group assignment [133]. When possible, we will record the reasons for discontinuing participation, properly annotate them in the CONSORT diagram, and retain the data for potential exploratory subgroup analyses.

All outcome models adjust for a pre-specified set of covariates, determined a priori on substantive grounds rather than on observed baseline balance. For each outcome, the primary ANCOVA includes the baseline (T1) value of that outcome as a covariate. Additionally, analyses for inflammatory markers will control for variables known to influence inflammation (e.g., BMI). In keeping with our preregistered analysis plan and CONSORT guidance, covariates are held fixed across analyses regardless of any observed baseline differences between conditions. All primary and secondary outcomes are continuous and assessed at multiple time points. The primary hypothesis concerns the between-condition difference in each outcome at the post-intervention endpoint. Accordingly, the primary analysis will be an ANCOVA comparing conditions (BEST vs. WLC) for each outcome at the post-intervention endpoint, adjusting for baseline (T1) values. ANCOVA is specified a priori as the primary model based on the study design. Covariates are pre-specified as described above. Perceived stress is the single confirmatory primary outcome and is tested at α = 0.05, requiring no adjustment for multiplicity. The secondary outcomes (burnout, depressive symptoms, psychological well-being, sleep quality, and inflammatory biomarkers) are pre-specified but interpreted as exploratory. These analyses will provide effect-size estimates and are reported without formal confirmatory claims or adjustment for multiple comparisons, informing future adequately powered trials rather than definitively testing efficacy.

We anticipate approximately 19% attrition based on comparable mindfulness-based intervention trials [126], and the extent and reasons for missing outcome data will be reported by condition in the CONSORT flow diagram. Our analyses assume that data are missing at random (MAR) conditional on baseline covariates and observed outcomes, an assumption supported by the collection of baseline and intermediate measures likely to predict missingness. To maintain the ITT principle under this assumption, missing endpoint data will be addressed using multiple imputation [134], with ANCOVA estimates pooled across imputed datasets according to Rubin’s rules [135].

As a complementary, pre-specified sensitivity analysis under a missing-at-random assumption, a linear mixed model will be fit using all available data from all randomized participants with at least one post-baseline observation, with fixed effects for condition and time (categorical) and a random intercept for participants. The between-condition contrast at the post-intervention endpoint will be extracted from this model to correspond to the primary estimand, and change over time and the condition-by-time interaction will be examined as secondary, descriptive analyses [136,137]. As an additional sensitivity analysis, we will repeat the primary outcome models using a per-protocol approach, restricting to participants in the BEST condition who attended at least three of the six intervention sessions (and the corresponding WLC participants), to assess whether the intervention effect differs among those who received the intervention as intended. Since a per-protocol analysis does not preserve the original randomization, it will be interpreted as a complementary, exploratory analysis rather than a confirmatory test of the ITT result. Lastly, because the analyzable sample is modest, the trial is not powered for formal moderation or subgroup analyses. Any examination of whether intervention effects differ across physician subgroups (e.g., specialty) will be exploratory and hypothesis-generating, intended to inform future adequately powered trials rather than to provide confirmatory tests. All tests, primary and secondary outcomes, will be two-sided with an alpha level of 0.05. All analyses will be conducted in R version 4.4.3 [71].

*Mediation analyses.* Mediation analyses will be conducted using the PROCESS macro in R [138]. To enhance the temporal precedence of the intervention’s effect on perceived stress through each mediator, we will assess the outcome (perceived stress) and mediators (compassion, positive emotions, equanimity/mindfulness) for all participants at the midpoint of the six-week intervention (T2). We will fit a parallel mediation model that includes group assignment (BEST vs. WLC) at T1 as the predictor variable, compassion, positive emotions, and equanimity/mindfulness at T2 as the mediators, and PSS scores at T3 as the outcome. The model will control for T1 perceived stress and for each mediator’s T1 values.

Based on recommendations by MacKinnon et al. [139], we will test the significance of the indirect effects using a non-parametric bootstrap approach (5,000 samples), which yields an average point estimate and a 95% confidence interval (CI) for the indirect, direct, and total effects. The effect is considered significant if the CI does not include zero [138].

### Data management and monitoring

Data will be collected using Qualtrics, with access restricted to the principal investigators. This limited access heightens data security, minimizes data entry errors, and maintains study integrity. Additionally, participant confidentiality will be maintained by using de-identified data in Qualtrics. Given the low risk of serious adverse events and the limited follow-up window, a data monitoring committee (DMC) will not be utilized. The principal investigators, in collaboration with the IRB, will oversee the trial and ensure participant safety.

## Discussion

To our knowledge, this is the first trial to test the effects of a mindfulness intervention that includes all four components of the traditional Four Immeasurables practice. Findings from this trial will provide unique and valuable insights into the impact of a novel mindfulness intervention for an at-risk group of individuals who currently lack effective, widely available, and scalable practices to address the stress inherent to their occupation. If effective, results may suggest the utility of the BEST intervention for other stressed populations beyond healthcare providers.

From a traditional Buddhist perspective, cultivating the Four Immeasurables is an essential step toward liberation, or the ultimate goal of freedom from suffering [55]. The virtues of loving-kindness, compassion, sympathetic joy, and equanimity are in the spirit of benefiting others, fostering a mind inclined towards love, and counteracting feelings of anger, hatred, envy, attachment, and aversion [55]. From a psychological perspective, kindness, compassion, joy, and equanimity fall under the broader construct of positive emotional states, which have been shown to be associated with beneficial mental and physical health outcomes [140–144]. Given the importance of the emotional states cultivated through the Four Immeasurables practice from both traditional and psychological perspectives, and the common characteristics shared across the three highly prevalent symptoms of working in high-stress healthcare environments, namely reduced empathic concern and low positive affectivity, the Four Immeasurables practice may be uniquely positioned to support this at-risk group.

This study has several strengths, including the use of both subjective and objective measures of intervention effects, a randomized design, and institutional support to facilitate trial success. Several limitations should also be considered. First, this is an open-label trial. Given the nature of a mindfulness intervention, participants and intervention instructors cannot be blinded to condition [72,145,146], and participants are aware they are enrolled in a study of a meditation program, which may influence their responses to self-report measures. This concern is compounded by the use of a waitlist rather than an attention- or expectancy-matched active control. Since expectancy is not equalized across arms, intervention participants may anticipate benefit in a way waitlist participants do not, potentially inflating self-reported outcomes, a recognized limitation of waitlist-controlled designs. These expectancy and self-report biases are somewhat mitigated by our objective, inflammation-based outcome, which is more difficult for participants to influence than self-report measures.

A related limitation concerns allocation concealment. Because the principal investigator conducts eligibility screening, informed consent, and baseline assessment while also holding the master randomization list, allocation is not concealed through personnel separation. However, the randomization sequence was generated before enrollment, so individual assignments were fixed in advance and could not be altered. Therefore, concealment relied on adherence to the pre-specified procedure of not consulting assignments until after baseline completion, supported by predefined, largely objective eligibility criteria that limit the potential for foreknowledge to influence enrollment.

The principal investigator also conducts the analyses unblinded to allocation. Although an independent, blinded analyst was not feasible given the size and structure of the trial, this risk is mitigated by preregistering all primary and secondary outcomes and the analysis plan (ClinicalTrials.gov, NCT07283744, and OSF, osf.io/npcax), which constrains analytic flexibility. Analyses will adhere to the preregistered plan, and any deviations will be explicitly identified and justified.

Additionally, the trial’s sample size is also constrained by recruitment capacity, with an anticipated enrolled sample of 90 and projected attrition of approximately 19% [126]. The study is powered to detect a moderate between-group effect (*d* = 0.58), consistent with those typically observed for digitally delivered mindfulness-based interventions [124], but may not detect smaller true effects. Findings should therefore be interpreted as informative about moderate-magnitude effects rather than as definitive tests of smaller ones.

More broadly, our intervention targets individual-level psychological processes, whereas the literature on physician well-being consistently identifies organizational and structural factors—including workload, administrative and clerical burden, electronic health record demands, and scheduling—as major drivers of stress and burnout. BEST is not designed to modify these systemic factors, and our findings should be interpreted accordingly. Should the intervention demonstrate benefits, these effects should be understood as individual-level coping resources that complement, rather than substitute for, organizational efforts to improve provider well-being, and should not be taken to imply that responsibility for addressing burnout rests solely with physicians. Conversely, limited or null effects may reflect constraints imposed by an individual-level approach on organizationally generated stressors rather than the ineffectiveness of the practices themselves. We view individual- and organizational-level approaches as complementary and necessary components of a comprehensive strategy for physician well-being.

Finally, the optional post-intervention feedback form is completed only by intervention-arm participants who choose to respond. These data may therefore reflect self-selection and may not be representative of all participants. We treat these qualitative responses as exploratory rather than as a representative account of intervention acceptability.

### Dissemination

Findings from this trial will be disseminated through peer-reviewed journal manuscripts, academic conferences, and meetings with local, state, and national hospital administrators.

## Conclusion

In summary, this trial not only aims to reduce distress and enhance well-being in a vulnerable population but also to provide valuable empirical evidence to the growing body of literature on the utility of traditional meditative practices in contemporary settings. The intervention as a combined package is a relatively unexplored topic ripe for important scientific discoveries that could add to the burgeoning evidence base of positive psychological interventions.

## Supporting information

S1 Fig FIERCE SPIRIT 2025 checklist 03_16_2026. (DOCX)

S1 File FIERCE Protocol. (DOCX)
