# Supplementary material for: Protocol for project FIERCE: A randomized controlled trial to evaluate a positive emotion-focused meditation intervention for physicians with elevated stress
Source: PLoS One. 2026 Sep 9;21(9):e0357906. doi: 10.1371/journal.pone.0357906 (PMC13557368; doi:10.1371/journal.pone.0357906)
Supplement: Supplementary file 2 [file pone.0357906.s002.docx]

**Project F.I.E.R.C.E – Fostering Internal and External Respect, Compassion, and Equanimity: A Mindfulness-Based Intervention to Cultivate Loving-Kindness, Compassion, Joy, and Equanimity in Healthcare Professionals**

Principal Investigators: Tyrus Korecki, MA

Julienne Bower, PhD

**TABLE OF CONTENTS**

[1. STUDY DESIGN:	4](#_Toc209700359)

[2. STUDY AIMS:	4](#_Toc209700360)

[3. BACKGROUND:	5](#_Toc209700361)

[3.1 Healthcare Provider Stress and Its Consequences	5](#_Toc209700362)

[3.2 Interventions for Healthcare Providers	5](#_Toc209700363)

[3.3 Stress and Inflammation	6](#_Toc209700364)

[3.4 Mediators of MBIs and the Four Immeasurables Practice	7](#_Toc209700365)

[3.5 Rationale for Proposed Study	8](#_Toc209700366)

[4. PARTICIPANT SELECTION:	9](#_Toc209700367)

[4.1 Inclusion Criteria.	9](#_Toc209700368)

[4.2 Exclusion Criteria.	9](#_Toc209700369)

[5. PARTICIPANT RECRUITMENT, ELIGIBILITY SCREENING, AND ENROLLMENT PROCEDURES	10](#_Toc209700370)

[5.1 Recruitment of Healthcare Providers	10](#_Toc209700371)

[5.2 Procedures and Study Flow	10](#_Toc209700372)

[*Eligibility Screening, Consent, Enrollment, and Baseline Assessment (T1)*	10](#_Toc209700373)

[*Randomization*	11](#_Toc209700374)

[*Mid-Course Assessment (T2).*	11](#_Toc209700375)

[*Post-Intervention Assessment (T3).*	11](#_Toc209700376)

[5.3 Financial Considerations for Participants	11](#_Toc209700377)

[5.4 Study Team Training	11](#_Toc209700378)

[6. STUDY INTERVENTION	12](#_Toc209700379)

[7. INTERVENTION FIDELITY, ADHERENCE, AND RELATED VARIABLES	13](#_Toc209700380)

[8. OUTCOME MEASUREMENTS	13](#_Toc209700381)

[9. INFORMATION ABOUT THE STUDY DATA	19](#_Toc209700382)

[10. DATA SAFETY MONITORING PLAN	19](#_Toc209700383)

[10.1 Risk/Benefit Assessment	19](#_Toc209700384)

[10.2 Data Monitoring and Security	21](#_Toc209700385)

[10.3 Safety Monitoring	21](#_Toc209700386)

[10.4 Monitoring Process	21](#_Toc209700387)

[10.5 Adverse Events/Stopping Rules	23](#_Toc209700388)

[10.6 Privacy and Confidentiality	23](#_Toc209700389)

[10.7 Data and/or Specimens for Future Research	24](#_Toc209700390)

[11. STATISTICAL ANALYSIS AND SAMPLE SIZE	24](#_Toc209700391)

[11.1 Sample Size	24](#_Toc209700392)

[11.2 Data Analysis Plan	25](#_Toc209700393)

[11.3 Analyses for Specific Aims and Hypotheses	27](#_Toc209700394)

[12. REFERENCES	29](#_Toc209700395)

[12. APPENDIX A	41](#_Toc209700396)

[13. APPENDIX B	42](#_Toc209700397)

# 1. STUDY DESIGN:

This single-site, two-arm, parallel-group RCT is designed to evaluate the feasibility and efficacy of a meditation-based intervention (Building Emotional Strength Training [BEST]) for perceived stress relative to a waitlist control for full-time healthcare workers. The intervention will be delivered by an experienced mediation instructor, following a manualized protocol, in a live online group format once a week for six consecutive weeks, held on Zoom. Groups will be conducted during the workday at standardized times. Assessments will be conducted both before and after the intervention to evaluate the effects of the intervention on the outcome.

Study participants will be 80 full-time healthcare workers employed by UCLA Health. They will be recruited in cohorts of 20-30 and randomized 1:1 to one of the two study arms. Participants will complete questionnaires and undergo blood collection on their own, which will be conducted before and after the six-week intervention. Additionally, several mechanisms of intervention effects will be assessed at the midpoint of the intervention (week three of the six-week intervention). Following CONSORT guidelines, we have designated one primary outcome for this trial: perceived stress. Additional (secondary) outcomes include symptoms of burnout, depression, subjective sleep quality, psychological well-being, and inflammatory processes, all of which have been associated with healthcare provider health and well-being and have been shown to be sensitive to the effects of meditation interventions. Further, several theory-based mechanisms of intervention effects include self-compassion, compassion for others, positive and negative emotions, equanimity, and mindfulness, all of which are plausible pathways through which meditation-based interventions may influence perceived stress.

# 2. STUDY AIMS:

**Aim 1:** Assess the feasibility of a mindfulness-based intervention based on the traditional Four Immeasurables practice for healthcare workers.

**Aim 2:** Determine the effects of BEST vs. waitlist control on perceptions of stress. We hypothesize that the BEST intervention, compared to a waitlist control, will result in significant reductions in perceived stress (primary outcome) among at-risk healthcare workers.

**Aim 3:** Evaluate the effects of BEST vs. waitlist control on negative and positive psychosocial symptoms experienced by healthcare workers. We predict that the BEST intervention will result in decreases in burnout and depressive symptoms and increases in psychological well-being and sleep quality (secondary outcomes) compared to a waitlist control.

**Aim 4:** Determine the effects of BEST vs. waitlist control on inflammatory processes associated with stress and relevant for long-term health. We predict that the BEST intervention will reduce markers of inflammatory activity at the genomic and systemic levels (secondary outcomes).

**Aim 5:** Explore theoretically based mechanisms of the effect of the BEST intervention on the primary outcome, perceived stress (exploratory aim). We hypothesize that the BEST intervention will enhance compassion for oneself and others, promote positive emotions, foster equanimity, and cultivate mindfulness, and that improvements in these variables will account for the intervention’s effects on perceptions of stress. Based on several theoretical models of mindfulness and kindness/compassion-based interventions, compassion (self and other), positive emotions, equanimity, and mindfulness are plausible pathways through which these interventions influence relevant outcomes (e.g., perceptions of stress).

# 3. BACKGROUND:

## 3.1 Healthcare Provider Stress and Its Consequences

Healthcare providers face a large number of stressful situations daily, which may increase their risk of developing several adverse mental and physical health conditions (1–4). Some of the most common complaints and downstream consequences of working in high-stress healthcare occupations are elevated levels of perceptions of stress, depression, and burnout. Each of these conditions has been associated with a host of unfavorable occupational outcomes, including absenteeism (5–7), lost work productivity (8,9), increased medical errors (10–13), and poorer patient quality of care (14–17). Additionally, perceived stress, depression, and burnout have been associated with adverse provider outcomes, including increased rates of suicidality (18–20), substance misuse (18,21), exhaustion (22,23), musculoskeletal pain (24), and gastrointestinal complaints (24–26). Finally, these conditions impose a significant financial burden on an already strained healthcare system in the form of worker turnover (5,27,28) and medical malpractice (12,29).

One of the common components shared between these conditions that may be particularly detrimental in a caregiving environment is an unsympathetic, cold, and detached demeanor toward one’s patients. Deficits in empathic concern may hinder a healthcare provider’s ability to provide adequate care to patients in need (30). With the high prevalence of elevated perceptions of stress, depression, and burnout among healthcare workers, the adverse patient and provider-related outcomes associated with these conditions, and the staggering costs imposed on society, there is a clear need for effective treatments to buffer against the adverse effects of healthcare provider stress.

## 3.2 Interventions for Healthcare Providers

To date, several attempts have been made to address psychological distress among healthcare providers, with mixed results (31–34). There is considerable variability in treatment approaches and intervention targets among individual-focused interventions designed to reduce distress and enhance well-being in this population. Meta-analytic data suggest that many interventions have short-lived treatment effects (35), high variability in effectiveness across different treatment approaches (36), and low attendance rates (ranging from 36% to 65%; 37–39), indicating a need to investigate alternative options for this at-risk group.

One group of interventions that may prove to be beneficial for reducing distress and enhancing well-being in healthcare workers is mindfulness-based interventions. While a variety of mindfulness-based interventions appear to be an acceptable and feasible option for stressed populations (e.g., Mindfulness-Based Stress Reduction (35) and Mindfulness-Based Cognitive Therapy (36)), there may be utility in offering meditation-focused interventions to healthcare providers that specifically aim to enhance prosocial sentiments and positive emotions. One such intervention is the Four Immeasurables, also known as the Brahmaviharas in Pali.

The Four Immeasurables were described in the Fifth Century in one of the primary treatises on Buddhist practice, the Visuddhimagga (37), and include the distinct emotional qualities of loving-kindness, compassion, sympathetic joy, and equanimity (38). These emotional states are gradually cultivated through specific practices that include visualizing oneself and others and directing the feeling state of the particular component (e.g., loving-kindness) to one’s target through the recitation of phrases such as “may you live with ease.” The practitioner is instructed to begin cultivating the emotional state by starting with an easy target (traditionally, the self, but it can be a benefactor if the self is too difficult). As the emotional state becomes more accessible and easier to elicit, practitioners progressively expand the scope of recipients, eventually directing the states to all living beings.

While there is a shortage of trials testing the effects of the traditional Four Immeasurables practice, empirical evidence suggests that mindfulness interventions that include exercises designed to cultivate loving-kindness, compassion, joy, and equanimity beneficially impact psychological, social, and biological outcomes (39–45). Additionally, these four positive psychological constructs may be particularly beneficial in lessening the common occupational-related symptoms experienced by healthcare providers, namely perceived stress, depression, and burnout. Furthermore, the favorable social influences that the Four Immeasurables appear to foster may suggest that these practices would significantly benefit individuals in caregiving occupations (i.e., healthcare workers), which could lead to improved patient outcomes and enhanced provider well-being.

## 3.3 Stress and Inflammation

Growing evidence suggests that stress exposure can influence physical and mental health-related outcomes (e.g., cardiovascular disease, diabetes, depression, etc.) by stimulating pro-inflammatory biology (46–48). Additionally, inflammation can have adverse effects on cognitive, emotional, and behavioral outcomes (49–51), which could further impair the effectiveness of healthcare providers. Given the growing evidence implicating inflammation as a key pathway through which stress influences physiological and psychological outcomes relevant to healthcare providers and other stress-exposed populations, it is critical to determine if heightened inflammation can be targeted and reduced with the BEST intervention.

There is a theoretical and empirical basis through which mindfulness interventions may influence inflammatory biology. Theoretical models of mindfulness propose that mindfulness interventions may influence downstream physiological and psychological outcomes by modulating cognitive and emotional processing of and reactivity to stressful situations (52–55). Indeed, several studies have demonstrated that mindfulness training can reduce negative responses to stress (56–58). Additionally, mindfulness-based interventions have been shown to beneficially influence genomic markers of inflammation, including the pro-inflammatory transcription factor nuclear factor (NF)-κB (59,60), and circulating protein markers of inflammation, such as interleukin (IL)-6 (43,44,61) and C-reactive protein (CRP) (62). While there is a scarcity of trials examining the effects of mindfulness-based interventions on markers of inflammation in healthcare providers, there is evidence of beneficial intervention effects in general adult populations (63).

## 3.4 Mediators of MBIs and the Four Immeasurables Practice

Despite the excitement surrounding the use of MBIs, the psychological pathways that mediate their benefits are not well understood (64). Without knowing the specific targets of clinical change, MBIs may not be fully optimized to provide the most benefit to participants, thus leading to sub-optimal intervention outcomes (65). To optimize and advance the understanding of MBIs, investigators must determine how the practices bring about change by testing theory-relevant mediators in well-powered clinical trials (53,66). Furthermore, due to the heterogeneity of current interventions offered to healthcare providers, it is unclear which types of interventions are most beneficial (33). An investigation of theory-based mechanisms of the Four Immeasurables may identify particularly impactful intervention targets that could be leveraged to enhance future interventions.

The Four Immeasurables approach is hypothesized to reduce suffering and enhance well-being by targeting compassion (for oneself and others), joy/positive emotions, and equanimity/mindfulness. Thus, these processes are potential mediators of the intervention. From a Buddhist perspective, the first two pillars of the Four Immeasurables —loving-kindness and compassion — are closely tied to one another, and having one without the other is challenging. The 14^th^ Dalai Lama (67) says that “genuine compassion must have both wisdom and loving-kindness…one must understand the nature of the suffering from which we wish to free others (wisdom), and one must experience deep intimacy and empathy with other sentient beings (loving-kindness).” Given this interrelationship, the path through which the loving-kindness and compassion practices work to reduce suffering and improve well-being is likely by enhancing experiences of compassion for oneself and others.

The third pillar of the Four Immeasurables – sympathetic joy – is an unadulterated delight in the happiness of others, which leads the observer to experience the same level of happiness as the observed individual. The ultimate “goal” of the sympathetic joy practice is that the practitioner arrives at a place where the feelings of joy are just as intense in response to the good fortune of others as they would be if the event had happened to oneself. Thus, the primary consequence of identifying and appreciating the positive emotional state of others is the equal experience of joy in the observer. Therefore, the pathway through which the sympathetic joy practice influences relevant outcomes is likely to be through increased experiences of positive emotional states.

The final pillar of the Four Immeasurables – equanimity - is a calm, impartial, evenness of mind and heart that approaches every situation without bias or preconceived notions. The equanimity practice aims to cultivate an understanding that, although we wish for the suffering of others to end or for their good fortune to continue, there is only so much we can do, as their situation is ultimately defined by their past and present thoughts and actions. The calm and accepting attitude that the equanimity practice cultivates is a component of many psychological definitions of mindfulness. Therefore, a reasonable route through which the equanimity practice works to reduce distress and enhance well-being is through improvements in equanimity/mindfulness.

These four variables represent the targets of change for the four distinct practices offered during the Four Immeasurables practice. Additionally, all four components (or their psychological correlates) have been shown to be inversely associated with adverse psychological states (68–74). Therefore, the Four Immeasurables practice may influence compassion (for self and others), positive emotions, and equanimity/mindfulness, and changes in these variables will serve as pathways through which the Four Immeasurables influence relevant outcomes (e.g., perceived stress).

## 3.5 Rationale for Proposed Study

There is a critical need to develop effective interventions to support individuals in healthcare occupations whose primary tasks involve repeated exposure to stressful, intense, and potentially traumatic events, as well as regular patient interaction (75,76). Considering the unique challenges faced by healthcare workers, the common psychological symptoms experienced by those individuals, and drawing from the theoretical and empirical literature on mindfulness-based interventions, the present study aims to test the feasibility and acceptability of a six-week, group-based mindfulness intervention inspired by the traditional Four Immeasurables practice. Empirical evidence suggests that loving-kindness, compassion, joy, and equanimity beneficially impact psychological and social outcomes (39–41). Additionally, these four positive psychological constructs may be particularly beneficial in lessening the common occupation-related symptoms experienced by healthcare providers, namely perceptions of stress, depression, and burnout. Furthermore, the beneficial social influences that the Four Immeasurables appear to foster may suggest that these practices would significantly benefit individuals in caregiving occupations (i.e., healthcare workers), which could lead to improved patient outcomes and enhanced provider well-being.

The design of the intervention was inspired by the traditional Four Immeasurables practice that has been a pillar of Buddhist teachings since the Fifth Century (37), as well as several secularized mindfulness interventions delivered in clinical and non-clinical settings for the reduction of psychological distress (77,78) and the promotion of well-being (79). To develop the intervention, we enlisted the assistance of an experienced meditation teacher, Diana Winston, to create a six-week mindfulness intervention centered on the traditional Four Immeasurables practice. Ms. Winston is the director of UCLA Mindful and has decades of experience working with novice meditators and creating secularized mindfulness programs for general populations (e.g., the Mindful Awareness Practices program).

The primary outcome of the study is perceived stress, given the high prevalence of elevated perceptions of stress among healthcare providers (3) and the adverse provider, patient, and organizational outcomes associated with elevated levels of provider stress (7,18,21). Secondary outcomes will include psychological (depression, burnout, and psychological well-being), behavioral (sleep quality), and biological (markers of inflammation) variables that have also been identified in healthcare providers and may be influenced by mindfulness-based approaches. Mediators will include compassion for self and others, positive emotions, and equanimity/mindfulness.

One of the most common complaints experienced by healthcare providers is elevated levels of depressive symptoms (27,80). Additionally, elevated depressive symptoms have been associated with several adverse patient and provider-based outcomes (10,11,19). Mindfulness-based interventions have been shown to be a safe and effective alternative treatment for the reduction of depressive symptoms (77,81). Given the high prevalence of heightened depressive symptoms in this population and the beneficial effects of mindfulness-based interventions on reducing depression symptom severity, depressive symptoms are a relevant secondary outcome.

Given the high rates of burnout among healthcare professionals (82) and its association with a host of unfavorable occupational (7,8,10,12) and patient-related outcomes (14–16), burnout is a logical and theoretically relevant outcome for this trial. Similarly, the psychological well-being of healthcare providers has been associated with several provider-related outcomes (e.g., job satisfaction, improved quality of care) (78,79) and patient-related outcomes (e.g., patient satisfaction, patient safety) (83,84). Additionally, given the focus of the Four Immeasurables practice (i.e., cultivating positive emotional states), psychological well-being is also a theoretically grounded and reasonable outcome.

Due to the nature of working in a healthcare setting, nearly 50% of healthcare providers consistently report sleeping poorly (85–87). Additionally, one of the hallmark symptoms of depression is dysregulated sleep (i.e., sleeping too little or too much) (88,89). Further, growing evidence suggests that mindfulness-based interventions can improve sleep quality in both clinical and non-clinical populations (90,91). Given the high frequency of poor sleep among healthcare providers, the close relationship between depressive symptoms and sleep quality, and the potential beneficial effects of mindfulness interventions on sleep-related outcomes, sleep quality is a crucial outcome to assess in this trial.

Finally, stress exposure, including occupational-related stress, negatively impacts mental and physical health, in part through upregulating inflammatory processes. Additionally, growing evidence suggests that mindfulness interventions may influence markers of inflammation, highlighting the importance of assessing this outcome in the trial.

To address the needs of this at-risk population, we propose to conduct a two-arm, parallel-group RCT to test the efficacy of the group-based, six-week BEST intervention compared to a waitlist control. Effects will be assessed at post-intervention. Additionally, theoretically based mediators of intervention effects will be evaluated at the midpoint of the intervention to determine the pathways through which BEST influences our primary outcome. If effective, the BEST intervention could provide a standardized and easily disseminated program to reduce stress-related symptoms in vulnerable healthcare providers and other stress-exposed populations.

# 4. PARTICIPANT SELECTION:

4.1 Inclusion Criteria. 1) full-time physician (M.D. or D.O.), 2) at least 18 years of age, 3) able to attend the six-week intervention, 4) fluent in English, and 5) access to the internet and email.

4.2 Exclusion Criteria. 1) having a current and consistent meditation practice (i.e., engaging in a formal meditation practice at least three days per week for five minutes each day), 2) previous participation in a Four Immeasurable course (e.g., attending a Brahmaviharas meditation retreat), 3) absence of at least mild levels of perceived stress as determined by a score less than four on the four-item Perceived Stress Scale (PSS-4; 92), 4) regular tobacco use (given its impact on immune function; 90), and 5) failure to agree to the informed consent. The presence or absence of perceived stress will be assessed using the PSS-4 during the initial phone screening. Given our primary outcome of perceptions of stress, we will exclude participants who do not experience at least mild levels of stress.

# 5. PARTICIPANT RECRUITMENT, ELIGIBILITY SCREENING, AND ENROLLMENT PROCEDURES

## 5.1 Recruitment of Healthcare Providers

Relationships with senior staff members of several departments within the UCLA Health System (e.g., Division of General Internal Medicine) will be leveraged to identify and recruit potential participants. Diana Winston, Director of UCLA Mindful, has enlisted the assistance of several healthcare professionals at UCLA who focus on the health and well-being of the healthcare employees in the UCLA Health system. The research team has spoken with Dr. Evelyn Curls, Vice Chair of Ambulatory Medicine for the Department of Medicine and Clinical Chief of the UCLA Division of General Internal Medicine and Health Services Research, Dr. Sun Yoo, Medical Director of UCLA Health Internal Medicine Suites, Dr. Elizabeth Ko, Medical Director of the UCLA Health Integrative Medicine Collaborative, and Liezl Meyer, BSN, RN, facilitator of the UCLA Health Nursing wellness program, about the project. These individuals are willing to leverage their networks to help advertise the project and facilitate participation (e.g., adjusting work schedules so that clinicians have dedicated time built into their schedules to attend intervention classes). The aforementioned senior staff members at UCLA have agreed to assist with recruitment by allowing us to email the recruitment flyer to potential participants under their supervision (e.g., General Internal Medicine, UCLA Health Integrative Medicine Collaborative) via their organizational listservs at UCLA Health. Further, other recruitment sources will be explored, including 1) announcements at staff meetings, 2) posting study fliers around the UCLA Health campus, 3) attendance and announcements at provider wellness events (e.g., Reigniting the Spirit of Caring program), and 4) advertisements in provider-focused newsletters (e.g., Wellness Wednesday and UCLA Health Vital Signs). All advertisements will direct interested individuals to contact the study staff via the email listed on the recruitment flyer.

## 5.2 Procedures and Study Flow

*Eligibility Screening, Consent, Enrollment, and Baseline Assessment (T1).* Interested participants who saw the recruitment flyer or heard about the study during various announcements at provider events (e.g., staff meetings, wellness events, etc.) will contact the study staff via email. A study staff member will respond to inquiry emails to schedule a call with the interested individual to provide additional information about the study, conduct the eligibility screening, and complete the study consent process.

Research staff will contact participants by phone at the scheduled appointment time to confirm their eligibility for the study. All participants who are determined to be eligible will then review and verbally acknowledge the IRB-approved consent form. Consent will be obtained verbally immediately following the eligibility screening during the same phone conversation. The same study staff member who completed the eligibility screening with the participant will immediately transition from the eligibility screening form/script to the IRB-approved consent script/form. The study staff member will review the consent form with the participant, obtain verbal consent, and then email a copy of the completed consent form to the participant immediately after the phone conversation. If an eligible participant does not have sufficient time to review the study consent form immediately following the eligibility screening, a follow-up phone call will be scheduled to complete the consent process.

After obtaining consent and enrollment, participants will be emailed a Qualtrics link to complete the study questionnaires. They will also receive an at-home blood collection kit in the mail to provide a blood sample for immune evaluation. Participants will receive a detailed set of instructions on when and how to complete the dried blood spot collection. Baseline assessments will be conducted during the 2 weeks preceding the onset of intervention. Study staff completing the eligibility screening, consent, and emailing participants the assessment links will be blind to participant group assignment.

Participants will be recruited in cohorts of 20-30 healthcare providers, who will be randomly assigned to either the BEST group or the waitlist control, allowing for our desired mindfulness group size of 10–15 participants. Screening will continue until we have an adequate number of eligible, interested, and available participants for the next cohort.

*Randomization.* Upon completion of the baseline assessment, participants will be randomized 1:1 to one of the two study conditions using the Qualtrics randomization feature by the study coordinator. The study coordinator will inform participants of their assigned condition and provide them with information on the intervention (e.g., class dates and times, Zoom link, etc.).

*Mid-Course Assessment (T2).* At the halfway point of the intervention, all participants will be emailed a link to complete self-report questionnaires assessing the primary outcome and mediator variables only.

*Post-Intervention Assessment (T3).* All participants will be emailed the post-intervention assessment link and mailed a second blood collection kit to complete both tasks within 2 weeks following the conclusion of the study intervention. The questionnaire will include all study variables.

See Appendix A for a visual description of the study procedures.

## 5.3 Financial Considerations for Participants

Participants will not be paid for their participation. Additionally, neither participants nor their insurers will be billed for any protocol-required services or investigational products.

## 5.4 Study Team Training

All study team members will receive adequate training and be informed about the research procedures and requirements. Team members will review the study protocol and all relevant documents (e.g., the informed consent form) and have the opportunity to ask questions to clarify any confusion. Additionally, all team members will practice the protocol procedures with other study team members before interacting with study participants. Further, team members will meet weekly to discuss procedures and relay any updates or changes to the protocol.

# 6. STUDY INTERVENTION

*General Information.* The intervention will be delivered remotely via Zoom. Group sessions will be conducted during the workday (around lunchtime).

*Building Emotional Strength Training (BEST).* The experimental mindfulness intervention was designed as a secularized version of the traditional Four Immeasurables practice. Diana Winston, Director of UCLA Mindful, developed the intervention based on her decades of experience working with new meditation practitioners and her expertise in designing group-based mindfulness courses for various audiences (94). In particular, she adapted a class previously offered through the Mindful Awareness Research Center (MARC) that focused on positive affect. Notably, the research team consulted with several healthcare professionals at UCLA to determine the most suitable delivery format, intervention duration, and optimal time of day for holding the intervention classes.

As reviewed above, the traditional Four Immeasurables practice includes exercises to promote the distinct emotional states of loving-kindness, compassion, sympathetic joy, and equanimity. The BEST curriculum is a six-week, group-based program where participants attend one class per week for six consecutive weeks. Each session focuses on a different component of the Four Immeasurables practice. During the one-hour class, participants will receive structured training and instruction in mindfulness exercises. The instructor will provide theoretical information and formal instructions on practicing the day’s specific topic and will allow students to ask questions to enhance their learning.

Additionally, participants will be guided through each meditation exercise introduced during class. Furthermore, each class will include dyadic discussions, allowing participants to interact directly with one another. The sessions will introduce the following topics and associated practices: 1) introduction to mindfulness, 2) further development of mindfulness, 3) loving-kindness, 4) compassion, 5) joy, and 6) equanimity.

The BEST intervention also includes home practice, in which participants are encouraged to practice the meditation technique introduced in class that week at home, starting with five minutes and gradually increasing to 20 minutes per day throughout the six weeks of the intervention. Diana Winston will teach all classes.

*Waitlist Control* *(WLC).* The WLC will control for naturally occurring changes in the outcomes and mediators over the assessment period. Participants in this group will not engage in any study-related activities outside of the remote assessments. Participants in the WLC will only complete the baseline assessment (T1; self-report questionnaires and at-home blood collection), the mid-course assessment (T2; self-report questionnaires) during week 3, and the post-intervention assessment (T3; self-report questionnaires and at-home blood collection) within 2 weeks following the conclusion of the study intervention. Participants randomized to this condition will be offered the BEST intervention after all data collection is completed.

# 7. INTERVENTION FIDELITY, ADHERENCE, AND RELATED VARIABLES

*Fidelity.* Fidelity to the protocol for the BEST condition will be ensured through the careful design and manualization of the intervention, as well as the monitoring of intervention delivery and receipt. Best practices from the Treatment Fidelity Workgroup of the NIH Behavior Change Consortium will be followed (95).

*Adherence.* We will track attendance at intervention group meetings. Participants in the BEST condition will be asked to record the frequency and duration of meditation practice between classes using a homework log.

*Acceptability.* The acceptability of the intervention will be evaluated using an abbreviated and adapted version of the Treatment Satisfaction Questionnaire (TSQ) (96), administered at the post-intervention assessment (T3). The three-item TSQ determines the extent to which the participant views the intervention as helpful and beneficial (96). Respondents respond to three statements assessing their agreement and general perception of the intervention on an eight-point Likert scale, ranging from 0 (not at all) to 7 (extremely). Example items include “The intervention was useful for me” and “The intervention improved my quality of life.” All three items are summed to provide a total satisfaction score. The possible range of scores is 0–21, with higher scores indicating greater satisfaction with the intervention.

*Optional Post-Course Evaluation.* Following the completion of the six-week BEST program, participants randomized to the intervention will be asked to complete an optional course evaluation form. The form will be emailed to all participants randomized to the intervention and will explain the purpose of the evaluation form. The evaluation sheet will ask participants about various aspects of the program (e.g., recommendations for content modifications) and their overall impressions of the course content. Responses will be used by the course creator (Diana Winston) to improve the course in future iterations. The evaluation form will take no more than 10 minutes to complete and is completely voluntary.

# 8. OUTCOME MEASUREMENTS

Reliable, valid, and commonly used self-report questionnaires will be administered at baseline (T1), midcourse (T2), and post-intervention (T3) to assess participant characteristics and outcomes. All measures have been validated in adult populations and have been shown to be sensitive to the effects of mindfulness interventions. Questionnaires will be administered using Qualtrics. The list of measures is presented in Table 1.

*Demographics and occupational-related variables*. Demographics, including age, sex, race/ethnicity, income, educational status, relationship status, and employment status, will be collected at baseline. Occupationally related variables, including healthcare unit (internal medicine, emergency department, intensive care unit, labor and delivery, pediatric, oncology/hematology, orthopedics, post-anesthesia care unit), will be collected at baseline, along with the shift typically worked (i.e., day shift, evening shift, or night shift).

*Primary outcome: Stress.* Stress will be assessed using the ten-item Perceived Stress Scale (PSS) (92). The PSS evaluates how often individuals appraised situations in their lives as stressful during the past month (e.g., “In the last month, how often have you been angered because of things that were outside of your control?”). Respondents respond on a five-point Likert scale from 0 (never) to 4 (very often). All scores are summed to provide a total stress score. Scores range from 0 to 40, with higher scores indicating a higher level of perceived stress. The PSS has been widely used with healthcare professional populations (97,98) and has been shown to be responsive to mindfulness-based interventions (99).

*Secondary outcomes.* Secondary outcomes include depression, burnout, psychological well-being, sleep quality, and markers of inflammation. They will be assessed at baseline (T1) and at the post-intervention time point (T3).

*Depressive symptoms*. Depressive symptoms will be based on scores on the 20-item Center for Epidemiologic Studies-Depression Scale (CES-D), a commonly used depression measure (100). The CES-D is a reliable, validated, and widely used measure that includes central components of depressive symptomatology, including depressed mood, worthlessness, hopelessness, loss of appetite, and psychomotor retardation (100). Respondents respond to twenty statements that assess how often the individual felt or behaved during the past week (e.g., “I felt that I could not shake off the blues even with help from my family and friends”) on a four-point Likert scale from 0 (rarely or none of the time [less than 1 day]) to 3 (most or all of the time [5-7 days]). All 20 items will be summed to provide a total score. The possible range of scores is 0-60, with higher scores indicating a greater frequency of depressive symptoms. The CES-D has frequently been used to assess depressive symptoms in healthcare workers (27,101) and has been shown to be responsive to mindfulness interventions (102).

*Burnout.* Burnout will be measured via the 22-item Maslach Burnout Inventory for Medical Personnel (MBI-M) (103). Items are written in a form that assesses the frequency of personal attitudes and feelings within the past month (103). The MBI-M measures three components of burnout: emotional exhaustion, depersonalization, and reduced personal accomplishment. Emotional exhaustion refers to feeling emotionally overextended and exhausted by one’s work. It is assessed with nine items (e.g., “I feel used up by the end of the day”). Depersonalization refers to an impersonal response toward the recipients of one’s services (e.g., the patients under one’s care). It is assessed with five items (e.g., “I have become more callous toward people since I took this job”). Personal accomplishment refers to feelings of competence and achievement in work-related pursuits. It is assessed with eight items (e.g., “I feel like I am making a real difference in the lives of the people I work with”). Items are scored on a 7-point Likert scale from 0 (never) to 6 (every day). Higher scores on the emotional exhaustion and depersonalization subscales correspond to a greater experience of burnout. The personal accomplishment subscale is positively worded; therefore, a one-item increase corresponds to a greater experience of work-related efficacy. The three subscales are considered separate and cannot be combined into a single total score (103). Each subscale will be summed, providing a total score for emotional exhaustion, depersonalization, and personal accomplishment. The MBI-M has been validated in several samples of healthcare workers (104,105), and the three-factor structure of the MBI has been confirmed in the same population (106,107). Additionally, the MBI-M has been shown to be influenced by psychosocial interventions (108).

*Psychological well-being.* Well-being will be measured using the 14-item Mental Health Continuum Short Form (MHC-SF) (109,110). The MHC-SF is a 14-item scale that measures three dimensions of psychological well-being: hedonic, eudaimonic-social, and eudaimonic–psychological. Hedonic well-being refers to pleasurable states characterized by the presence of positive and absence of negative affect (109). Hedonic well-being is measured via three items (e.g., “During the past month, how often did you feel happy?”). Eudaimonic social well-being refers to flourishing within a social context characterized by social coherence and actualization (111). Eudaimonic social well-being is assessed via five items (e.g., “During the past month, how often did you feel that you had something important to contribute to society?”). Eudaimonic psychological well-being refers to a sense of living a life of purpose, characterized by feelings of personal growth and self-actualization (109). Eudaimonic psychological well-being is assessed via six items (e.g., “During the past month, how often did you feel that your life had a sense of direction or meaning to it?”). Respondents respond to the fourteen items to assess how often they felt concerning the statements during the past month, using a six-point Likert scale ranging from 0 (never) to 5 (every day). All scores are summed to provide a total well-being score. The possible range of scores is 0-70, with higher scores indicating greater subjective well-being. To our knowledge, the MHC-SF has not been used in a dedicated sample of healthcare providers. However, it has been shown to be reliable and valid in various adult populations (110,112). Additionally, the MHC-SF has been shown to be sensitive to the effects of mindfulness interventions (113).

*Sleep quality.* Sleep quality will be assessed using the seven-item Insomnia Severity Index (ISI) (114). The ISI is a reliable and valid self-report measure that evaluates the severity and impact of insomnia over the past two weeks. Respondents are asked to respond to the seven items to assess how they relate to each statement over the past two weeks, using a five-point Likert scale ranging from 0 (not at all) to 4 (very much). Scores are summed to obtain a total score. The possible range of scores on the ISI is 0–28, with higher scores indicating more severe insomnia. Additionally, scores of 15 or higher are indicative of clinically significant insomnia. The ISI has been validated for use with healthcare professionals (115) and shown to be responsive to mindfulness interventions (116).

*Inflammation.* We will utilize an integrated assessment of inflammation that encompasses systemic levels of inflammatory markers, inflammatory gene expression, and promoter-based bioinformatics analyses of specific inflammation-related transcription factors. Each measurement level provides unique and complementary information, offering a comprehensive view of inflammatory activity.

*Circulating inflammatory markers.* Assays will focus on pro-inflammatory cytokines and markers of inflammation that are responsive to mind-body interventions (117,118). Meso Scale Discovery multiplex immunoassays will be used to assess five key pro- and anti-inflammatory cytokines (IL-6, IL-8, IL-10, TNF-α, and IFN-γ), and R&D Systems ELISA will assess C-reactive protein (CRP). Previous mindfulness studies have demonstrated decreases in CRP (119,120) and IL-6 (59,121).

Circulating levels of IL-6, IL-8, IL-10, TNF-α, and IFN-γ will be quantified using a V-PLEX Custom Human Cytokine Proinflammatory Panel on the Meso Scale Discovery (MSD) electrochemiluminescence platform and Discovery Workbench software (122); the assay lower limit is 0.06 pg/mL for IL-6, 0.07 pg/mL for IL-8, 0.04 pg/mL for IL-10, 0.04 pg/mL for TNF-α, and 0.37 pg/mL for IFN-γ. Circulating levels of CRP will be quantified using Human Quantikine ELISA (123); the assay's lower limit is 0.8 ng/mL. Values below the lower limit of detection (LLD) will be replaced with values halfway between 0 and the LLD. For example, values below the LLD of 0.8 ng/mL for CRP will be replaced with values of 0.4 ng/mL.

*Molecular analysis of pro-inflammatory gene expression and signaling.* Analyses will focus on the expression of a set of 19 pro-inflammatory, 28 Type I interferon response, and three antibody synthesis genes that have been shown to be upregulated in the context of chronic stress (124). Additionally, this set of genes, labeled the Conserved Transcriptional Response to Adversity (CTRA), has been shown to be responsive to mindfulness-based interventions (59,125,126). Additionally, we will assess pro-inflammatory gene expression at the molecular level using a novel bioinformatics approach. This approach utilizes genome-wide transcriptional profiling to identify genes that exhibit differential expression in leukocytes from participants randomized to the BEST group versus the waitlist control. Furthermore, the approach uses the Transcription Element Listening System (TELiS) transcription factor search engine to identify the activation of specific transcription control pathways, including nuclear factor kappa-light-chain-enhancer of activated B cells **(**NF-κB), activator protein-1 (AP-1), and cAMP-responsive element-binding protein (CREB) signaling, which are hypothesized to underlie increased inflammatory activity. To identify the cellular sources of differentially expressed genes, we will carry out Transcript Origin Analysis (127). Assays for genomic markers of inflammation will be conducted at the UCLA Social Genomics Core Laboratory under the supervision of Dr. Steve Cole.

*Procedure for blood collection, processing, and storage.* The Tasso M-20 device will be used to collect, store, and transport capillary blood for testing at the Social Genomics Core Laboratory. The Tasso is designed for use in non-clinical settings with adult populations. After collection, samples will be stored in a -20 Celsius freezer and analyzed in one batch after data collection is completed.

*Mediators.* Selected mediators include compassion for self and others, positive emotions, and equanimity/mindfulness. They will be assessed at baseline (T1), the study midpoint (T2), and post-intervention (T3).

*Compassion (self and others).* In the psychological literature, the most commonly employed measure of loving-kindness and compassion towards oneself is the Self-Compassion Scale (128), which includes the self-kindness and self-judgment subscales (the antithesis of self-compassion). This measure assesses one’s ability to provide kindness, compassion, and support to oneself, but fails to expand those feelings of goodwill to others. Given the absence of feelings of benevolence towards others, Pommier and colleagues (129) developed an analogous scale, the Compassion Scale, which assesses feelings of kindness, compassion, and support for others. The combination of these two measures effectively captures the traditional constructs of loving-kindness and compassion, the two skills developed in the first two pillars of the Four Immeasurables.

Self-compassion will be assessed using the 12-item Self-Compassion Scale-Short Form (SCS-SF) (128,130). The SCS-SF assesses an individual’s global tendency to show kindness, concern, and sympathy for one’s own suffering (130). The scale has six subscales with two items each: self-kindness (compassion for oneself during difficult moments), self-judgment (disapproval or intolerance of oneself), common humanity (appreciation of the universal nature of having flaws and inadequacies), isolation (experiencing failures alone), mindfulness (balance and poise during difficult moments), and over-identification (obsessing over negative feelings). Individuals answer how often they generally behave in a stated manner on a five-point Likert scale from 1 (almost never) to 5 (almost always) in response to the 12 items (e.g., “When I’m going through a very hard time, I give myself the caring and tenderness I need.”). The six items from the self-judgment, isolation, and overidentification subscales are reverse-scored (i.e., 1 = 5, 2 = 4, etc.). The mean of each subscale is then calculated to compute a total mean (the average of the six subscale means). Scores range from 1 – 5, with higher scores indicating greater feelings of self-compassion. The SCS-SF has been used in the context of an intervention for healthcare workers and demonstrated sensitivity to changes following the intervention (131).

Compassion for others will be assessed using the 16-item Compassion Scale (CS) (129). The CS comprises four subscales, each with four items, that determine how one generally reacts toward others. The four subscales include kindness (compassion and caring towards others during times of difficulty), common humanity (appreciation for the universal experience of pain), mindfulness (awareness of others and their experiences), and indifference (an unsympathetic demeanor towards the suffering of others). Individuals answer how often they generally behave in a stated manner on a five-point Likert scale from 1 (almost never) to 5 (almost always) in response to the 16 items (e.g., “If I see someone going through a difficult time, I try to be caring toward that person.”). The four items from the indifference subscale are reverse-scored (i.e., 1 = 5, 2 = 4, etc.). The mean of each subscale is then calculated to compute a total mean (the average of the six subscale means). Scores range from 1 – 5, with higher scores indicating greater feelings of compassion for others. The CS has been used in healthcare worker populations (132). To the best of our knowledge, the CS has not yet been used in an intervention setting.

*Positive and negative emotions.* Positive emotions will be assessed using the 20-item modified Differential Emotions Scale (mDES) (133). The mDES is based on Fredrickson’s Broaden-and-Build theory of positive emotions (39,134) and assesses the degree to which individuals experience a spectrum of discrete positive and negative emotions within a given timeframe. The questionnaire includes ten items assessing positive emotions, such as contentment, amusement, and awe, and ten items assessing negative emotions, such as sadness, shame, anger, and hatred. The mDES attempts to capture the experience of both high and low arousal positive and negative emotions (133). For each item, respondents indicate the extent to which they felt a specific emotion in the past 24 hours on a five-point Likert scale, ranging from 0 (not at all) to 4 (extremely). Each subscale (positive and negative) is independent of the other, so the mDES will yield a total score for both positive and negative emotions. Each subscale item is summed to provide total positive and negative emotion scores. Scores on each subscale range from 0 to 40, with higher scores on the positive subscale indicating a greater experience of positive emotions and higher scores on the negative subscale indicating a greater experience of negative emotions. The mDES has previously been used in healthcare populations (135) and has been shown to be influenced by mindfulness interventions (136,137).

*Equanimity.* Equanimity will be assessed using the 16-item Equanimity Scale (ES-16) (138). The ES-16 assesses the tendency of an individual to act in a balanced, calm, and even-tempered manner toward all experiences (positive or negative). The scale comprises two subscales, each consisting of eight items: experiential acceptance (neutrality toward all internal experiences) and non-reactivity (an evenness of response toward all experiences). Example items include, “When I experience distressing thoughts and images, I can accept the experience,” and “I can pay attention to what is happening in my body without disliking or wanting more of the feeling or sensation.” Individuals respond on a five-point Likert scale from 1 (strongly disagree) to 5 (strongly agree) to indicate how they view themselves. The eight items from the non-reactivity subscale are reverse-scored. All 16 items are summed to provide a total equanimity score. Scores range from 16 – 80, with higher scores indicating greater levels of equanimity. The scale has not been used with healthcare professionals, but it has been shown to be sensitive in detecting changes following a mindfulness intervention (139).

*Mindfulness.* Mindfulness will be assessed using the 15-item Five Factor Mindfulness Questionnaire (FFMQ-15) (140,141). The FFMQ assesses five distinct facets of mindfulness using five subscales: observing (e.g., “I pay attention to how my emotions affect my thoughts and behavior”), describing (e.g., “I can usually describe how I feel at the moment in considerable detail”), acting with awareness (reverse scored; e.g., “I am easily distracted”), non-judging (reverse scored; e.g., “I criticize myself for having irrational or inappropriate emotions”), and non-reactivity (“I perceive my feelings and emotions without having to react to them”). Individuals respond on a five-point Likert scale, ranging from 1 (never or very rarely true) to 5 (very often or always true), to indicate how each statement relates to their general attitude. All items are summed and divided by 15 to provide an average mindfulness score. Scores range from 1 – 5, with higher scores indicating greater mindfulness. The FFMQ has been utilized with healthcare workers (142) and has been demonstrated to be sensitive to the effects of mindfulness interventions in the same population (143).

| **Table 1. List of Measures** | **T1** | **T2** | **T3** |
| --- | --- | --- | --- |
| **Background characteristics** |  |  |  |
| Demographic characteristics | X |  |  |
| Employment | X |  |  |
| Health history, Use of mental health services | X |  | X |
| Height and weight measurements | X |  |  |
| Health behaviors/meds | X |  | X |
| Calendar Allocation and Continuing Medical Education Training |  |  | X |
| **Outcomes** |  |  |  |
| Perceived stress (Perceived Stress Scale [PSS]) | X | X | X |
| Burnout (Maslach Burnout Inventory for Medical Personnel [MBI-M]) | X |  | X |
| Psychological well-being (Mental Health Continuum Short Form [MHC-SF]) | X |  | X |
| Depressive symptoms (Center for Epidemiologic Studies - Depression [CES-D]) | X |  | X |
| Sleep quality (Insomnia Severity Index [ISI]) | X |  | X |
| Circulating inflammatory markers | X |  | X |
| Pro-inflammatory gene expression | X |  | X |
| **Mediators** |  |  |  |
| Self-compassion (Self-Compassion Scale-Short Form [SCS-SF]) | X | X | X |
| Compassion for others (Compassion Scale [CS]) | X | X | X |
| Positive and negative emotions (modified Differential Emotions Scale [mDES]) | X | X | X |
| Equanimity (Equanimity Scale [ES]) | X | X | X |
| Mindfulness (Five Factor Mindfulness Questionnaire [FFMQ]) | X | X | X |
| **Feasibility and Acceptability** |  |  |  |
| Acceptability (Treatment Satisfaction Questionnaire [TSQ]) |  |  | X |

# 9. INFORMATION ABOUT THE STUDY DATA

Data related to health or mental health care will be collected to characterize the study sample (see above). All data collected related to health or mental health will be used solely for study purposes and will not be used to inform health or mental health care decisions. The data will be collected via self-report, and medical records will not be accessed or updated with research data.

Participants' names, postal addresses, phone numbers, and email addresses will be collected during participation. Names and postal addresses will be used to send participants the dried blood spot collection kits. Phone numbers and email addresses will be used to contact participants for study-related activities (assessments, class reminders, etc.). The biospecimens will not be used for whole-genome sequencing.

The self-report data and blood samples collected will be coded using a study identification number that cannot be linked to individual study participants. They will be stored in a locked file cabinet or a password-protected computer in the research office or laboratory.

# 10. DATA SAFETY MONITORING PLAN

## 10.1 Risk/Benefit Assessment

This study involves a behavioral intervention targeting perceptions of stress among healthcare providers at UCLA Health and is considered a minimal-risk study. There are no study procedures that pose risks currently unforeseen.

*Potential Risks or discomforts associated with participation.* This study has minimal risk*.* The primary risks of study participation involve potential psychological distress related to the study questionnaires and/or intervention, burden/inconvenience secondary to the study assessments and interventions, discomfort with blood collection, and potential loss of confidentiality. The study questionnaires ask about mood and emotions. Although unlikely, given the widespread use and acceptability of the psychological measures included in the battery of assessments, answering some of these questions may cause minor discomfort to the participants. If discomfort is experienced while answering the questionnaires, it will likely be minor in nature, short-lived (i.e., lasting only a few moments), and not permanent. To mitigate this potential discomfort, participants can choose not to answer one or more of the questions on the study questionnaires.

Additionally, the mindfulness intervention includes discussions of emotional material, such as dealing with complex thoughts and feelings. Groups will be led by trained instructors who are experienced with handling these topics and the thoughts/feelings they may elicit.

During blood collection, participants may experience some pain, bruising, or bleeding due to the use of the Tasso device. They may also feel faint or nauseous during the dried blood spot collection, though these side effects are quite rare due to the small amount of blood drawn. There is also a rare risk of infection or allergic reaction. The probability that any of these adverse reactions will occur is small. We have utilized the Tasso device to collect dried blood spots in several studies, involving both clinical (e.g., breast cancer survivors) and non-clinical (e.g., undergraduate students) populations, and have not encountered any adverse events. The magnitude of discomfort is relatively low, would be short-lived, and reversible. The discomfort would be equivalent to accidentally pricking a finger with a safety pin. Given that all participants are licensed physicians, they will be well-equipped to perform the blood collection procedures safely and have the necessary medical knowledge to expertly respond to any discomfort that may arise. Still, to account for these discomforts, all blood collection will be performed using standardized procedures. Participants will be provided with step-by-step instructions and directed to watch a demonstration video to understand how to use the device safely before collecting any blood samples.

The amount of effort and time involved may be burdensome for some participants. The study has been designed to fit within busy care provider schedules. Study assessments will be conducted remotely, at times that are convenient to participants. Additionally, the intervention will be conducted virtually over Zoom to minimize travel time to and from group meetings.

Finally, to address risks related to confidentiality, we use only an identification number, and no other identifying information on all questionnaires and samples that have been/will be collected as a part of this study. Additionally, the data and samples are stored in a locked file cabinet or a password-protected computer in the research office or laboratory. In addition, the online web portal where questionnaires are administered will be password-protected. Questionnaire data will be stored on a secure server at UCLA and compiled separately from the web interface. Only members of the research team and the Investigators have access to the data. When the research results are published or discussed at conferences, no information will be included that would reveal the identities of study participants.

*Potential benefits associated with participation.* Mindfulness-based programs like the BEST program have been shown to reduce depressive symptoms, burnout, and stress and to increase well-being. Participants may experience these benefits after completing the program.

All risks, discomforts, and benefits are clearly outlined in the informed consent form and will be explained to participants during the consent process. Given the low-risk nature of the study procedures, the potential individual-level benefits to participating, and the possibility of benefiting a large number of individuals outside of this project by identifying an effective intervention to reduce common symptoms of this at-risk population, the benefits of conducting the project are reasonable in relation to the potential risks/discomforts.

## 10.2 Data Monitoring and Security

The Principal Investigators will be responsible for maintaining quality control over the data collection process and for securing its confidentiality. The Project Coordinator will oversee the data collection process, ensuring that data from self-report questionnaires and immune assays are reviewed for completeness and accuracy. The OHRPP Data Security in Research guidelines will be followed to ensure data security and participant confidentiality. In the event of a data security breach, the IRB and the Psychology Department's IT Compliance Coordinator will be notified immediately.

## 10.3 Safety Monitoring

The Principal Investigators will be responsible for participant safety in collaboration with the Project Coordinator and Intervention Instructor. The primary safety concern is for participants’ mental health, given our focus on participants working in high-stress occupations. We will take several actions to evaluate the mental status of participants:

1. The Project Coordinator will provide information on mental health resources at UCLA to all enrolled participants at the baseline assessment.

2. The Project Coordinator will monitor questionnaires completed by study participants at each assessment. Those who score 24 or above on the CES-D, which indicates severe depression, will be contacted and encouraged to seek out mental health services at UCLA Health.

3. Participants who volunteer information about suicidal thoughts or behaviors to study personnel (Project Coordinator, Intervention Instructor) will undergo immediate evaluation using the Columbia Suicide Severity Scale using a standardized study-specific protocol. Those who deny suicidal ideation or plans will be referred to mental health services at UCLA Health. Those who endorse suicidal ideation or plans will be asked to stay with study personnel who will contact mental health services for emergency evaluation and relevant action. In either case, study personnel will notify the Project Coordinator and Principal Investigators, who will then submit a report to the appropriate mental health services.

4. Participants can withdraw from the study at any time. They will be informed of their right to withdraw during the consent process. Given that participants will not be recruited from a clinical population and the low-risk, non-clinical nature of the intervention, there are no established guidelines for withdrawing participants.

## 10.4 Monitoring Process

Study performance and safety monitoring will be conducted. The following list outlines the items that will be regularly monitored and summarized every six months.

**General Information**

1. Results of the study to date; any interim (formal or informal) analysis. Interim analyses will include demographics of enrolled participants and intervention effects on primary and secondary outcomes (perceived stress and positive and negative psychosocial characteristics). All analyses will follow the statistical analysis approach outlined below.

2. All protocol amendments since the last submission, including any protocol modifications resulting from safety-related concerns.

3. Issues with study implementation.

4. A description of any protocol deviations since the last summary period. The description will include both purposeful and accidental variances in the approved procedures outlined in the protocol and provide the date reported to the IRB, as applicable.

5. Any abstract or publications derived from the study.

**Accrual Data**

1. Recruitment rates, including number of participants expressing interest, number screened, number eligible, number enrolled, and reasons for non-eligibility.

2. A description of any problems with recruitment or enrollment and a determination as to whether the current accrual rate is sufficient to meet the intended accrual goal.

3. Demographics of screened and enrolled participants by treatment group.

4. Adherence of participants to study treatment (i.e., attendance at classes).

5. Number of participants evaluated at each follow-up and reasons for losses to follow-up.

**Safety Data**

1. A list of all adverse events and their frequency. Adverse events will be categorized based on their relationship to the study intervention as “definitely,” “probably,” “possibly,” or “not related.”

**Un-blinding**

Given that assessments will be conducted remotely via self-report questionnaires, it is unlikely that a situation will arise where blinded study personnel will need to be unblinded. The only individuals who will have access to participant data (e.g., responses to self-report questionnaires) are the study coordinator and the PIs. These individuals will not be blinded to the participant group assignment and will not require unblinding.

**Individual Research Results**

Clinically actionable individual results are not anticipated. Participants will not be recruited from a clinical population; all data will be collected via self-report questionnaires and will not include assessments from a licensed clinician. The intervention is not designed to treat any mental or physical health condition. Therefore, the results from the study cannot be considered a valid or reliable clinical diagnosis and will not be shared with participants or their family members.

Participants will have the opportunity to receive general and overall results from the study at the conclusion of data analysis. These results will not include individual-level results and will only report on overall effects on primary and secondary outcomes. Participants can opt in or out of receiving these results during the consent process.

## 10.5 Adverse Events/Stopping Rules

Adverse events will be monitored during treatment and post-intervention to evaluate the presence of adverse events or serious adverse events related to the reduction of perceived stress in each group. Potential adverse events in this trial include severe levels of depressive symptoms, as determined by scores on the CES-D and/or reports of suicidal ideation, intention, or behavior. Additionally, we will monitor other adverse events, including illnesses and hospitalizations.

Based on the population being recruited and the low-risk, non-clinical nature of the intervention, it is unlikely that the trial would need to be stopped due to widespread adverse effects. Therefore, stopping rules have not been established.

## 10.6 Privacy and Confidentiality

Participant privacy and confidentiality are a top priority. Any information obtained in connection with this study that could identify participants will remain confidential. It will be disclosed only with the participant’s permission or as required by law. Confidentiality will be maintained by using only an identification number and no other identifying information on all questionnaires and samples collected as part of this study. The data and samples will be coded using a study identification number that cannot be linked to individual study participants, and they will be stored in a locked file cabinet or a password-protected computer in the research office or lab. Only members of the research team and the study investigators have access to the data collected. Additionally, when the research results are published or discussed at conferences, no information will be included that would reveal participant identities.

**Personally Identifiable Information (PII)**

All data and specimens collected throughout the study will be coded and will not be associated with any identifiable information (e.g., name, address, etc.). The study coordinator will assign a unique study ID to each participant. Online questionnaires will be collected online using Qualtrics. The “Anonymize Responses” option on Qualtrics will be enabled to strip the IP address from the questionnaire responses.

**Coded Data**

All electronic data will be securely stored using password protection software. Hardcopy data and specimens will be stored in a locked file cabinet and freezer within a locked laboratory, with access limited to authorized personnel (i.e., study staff members only). The code key will be securely kept in a separate room from the coded data/specimens in a locked file cabinet inside a locked office. After data collection is completed, the key to the code will be destroyed, and we will ensure that all personally identifiable information has been removed and destroyed.

Given the nature of the data being collected and the population participating, it is unlikely that any member of the research team will learn of any reportable information while conducting the research. Additionally, the study will not be covered by a Federal Certificate of Confidentiality (or equivalent protection). The consent form informs the participant about how their privacy and confidentiality will be maintained.

## 10.7 Data and/or Specimens for Future Research

Data collected from this study (self-report questionnaires and/or biological specimens) may be used for future research. Future uses may include, but are not limited to, the investigation of intervention effects on biological markers not included in the aims and outcomes of this study (e.g., effects on markers of biological age), investigations of biological or psychological mechanisms of intervention effects not already included in the present study, and investigations of moderators of treatment effects. The informed consent outlines the possible future use of data collected and allows participants to opt in or refuse the use of their data for future studies. If participants refuse, their data will be destroyed once all study aims and outcomes described here have been completed. If participants opt in, questionnaire data and biological samples will be stored for future use. All data will have been stripped of any direct or indirect identifiers.

Requests from other researchers to use any data collected will be reviewed on a case-by-case basis by the PIs. If a request is deemed appropriate, data collected via the self-report questionnaires and/or the biological specimens will be shared with other researchers. All data will have been stripped of any direct or indirect identifiers. The PIs will oversee and manage all data requests and the sharing of requested data.

There is a plan to retain participants' contact information for future studies. Participants have the opportunity to opt in or out of this option during the consent process. Participant name and email address will be retained to inform participants about the results of our study, notify them about the greater, positive impacts of the project (e.g., integration of the BEST program in standard care protocols at UCLA Health), and give them information about new research projects that they may potentially be eligible to participate in. The contact information will be limited to the study team.

# 11. STATISTICAL ANALYSIS AND SAMPLE SIZE

## 11.1 Sample Size

*Primary and secondary outcome analyses.* The study has been designed to provide 80% power to detect differences in the primary outcome, perceived stress, at the primary study endpoint (post-intervention) between the mindfulness condition and the WLC. Typical effect sizes from randomized controlled trials of mindfulness-based interventions with passive control groups range from *d*s = 0.10 – 0.89, with trials in healthcare populations achieving an average effect size of 0.39 and digitally delivered interventions achieving an average effect size of 0.54 (78). For example, a recent trial conducted by Aikens and colleagues (144) randomized employees at a large company to receive either a seven-week virtual, group-based mindfulness intervention (n = 44) or a waitlist control (n = 45). The mindfulness intervention consisted of one in-person introductory class and six one-hour virtual classes, which were held weekly for the remainder of the intervention. The authors found significant reductions in perceived stress and increases in mindfulness, resiliency, and vigor (average *d* = 0.67) (144).

We calculated the study’s sample size using the statistical software G*Power (145). For a priori sample size calculations of F-statistics, G*Power requires several inputs including, the effect size, alpha level, desired power, number of groups being compared, and either the numerator degrees of freedom, the number of assessment timepoints, or the correlation among repeated measures depending on the type of statistical test (e.g., ANOVA - fixed effects, main effects, and interactions, MANOVA – repeated measures, within-between interaction). We ran several different power analyses and report here on the most conservative results. The most conservative sample size utilized the ANOVA – fixed effects, main effects, and interaction statistical test in G*Power. We elected to use a conservative effect size of 0.40 based on our population and method of delivery. To achieve 80% power to detect small to medium effects (0.40) with a significance level of alpha = 0.05, two groups for comparison, and two degrees of freedom in the numerator, we need a total sample size of 64 (n = 32 per group). Results from all power analyses in G*Power are included in Appendix B.

To account for the ~20% attrition rate typically found in controlled mindfulness-based intervention studies (146), we will randomize 40 participants to each condition (total sample size: N = 80). The attrition rate is a conservative estimate based on a meta-analysis examining the overall and differential attrition rates across 114 randomized trials of MBIs in clinical and non-clinical populations (146). Power for secondary outcomes will be similar.

*Power for mediation analyses*. Meta-analytic data suggest that mindfulness-based interventions have a small to large effect on compassion, positive emotions, and mindfulness (*g*s = 0.07 – 0.8; (78,147–149). To calculate our achieved power for the mediation analyses, we used a conservative effect size of 0.30 for all the a and b paths and an effect size of 0.40 for the c’ path. Assuming a sample size of 80, our achieved power to detect indirect effects ranges from 73% to 78%.

## 11.2 Data Analysis Plan

*Descriptive analyses:* Descriptive statistics will be created to show the demographic and psychological characteristics of the sample. One-way analysis of variance (ANOVA) and chi-square tests will be conducted to examine baseline differences in demographic and occupation-related variables between the two conditions (mindfulness and WLC). If significant differences emerge, those baseline values will be included as covariates in outcome analyses. Additionally, we will examine the distributional properties of the study variables to assess normality. We will address issues of non-normality using applicable techniques, including robust standard errors and rescaling. It is common practice to log-transform inflammatory markers to normalize distributions.

*Feasibility and acceptability.* Feasibility will be evaluated based on participant attendance and engagement with course content (e.g., the amount of time spent practicing the meditative skills learned in class). Acceptability will be based on the Treatment Satisfaction Questionnaire (TSQ) scores. Analyses of the intervention’s feasibility and acceptability will be descriptive in nature.

*Primary and secondary outcomes.* All analyses will employ an intention-to-treat (ITT) principle. Thus, regardless of how many classes they attended or assessments they completed, all randomized participants will be included in analyses and analyzed based on their original group assignment (150). When possible, we will record the reasons for discontinuing participation, properly annotate them in the CONSORT diagram, and retain the data for potential exploratory subgroup analyses.

Given that all outcome measures are continuous and assessed at multiple time points, linear mixed models will be used to evaluate primary and secondary outcomes. Models will be fit using all available data, including data from participants with incomplete follow-up data, with fixed effects for condition and time and random effects for individuals (i.e., fixed slopes and random intercepts). Multilevel models are well-suited for analyzing repeated measurements and can provide unbiased model estimates, even in the presence of missing data (151,152). Differences between conditions over time will be tested with a condition-by-time interaction. If necessary, models will control for participant characteristics with baseline imbalances between conditions. Additionally, analyses for inflammatory markers will control for variables known to influence inflammation (e.g., BMI). All tests, primary and secondary outcomes, will be one-sided with an alpha level of 0.05. All analyses will be conducted in R version 4.4.3 (153).

*Mediation analyses.* Mediation analyses will be conducted using the PROCESS macro in R (154). The relationship between an outcome (Y), an explanatory variable (X), and a mediator (M) is characterized by four effects: the effect of X on M (a path), the effect of M on Y while controlling for X (b path), the total effect of X on Y (c path), and the effect of X on Y while controlling for M (c’ path). The mediated or indirect effect (the effect of X on Y through M) is characterized by the product of the a and b paths and represents the relationship of interest when testing mediation (154).

To enhance the temporal precedence of the intervention’s effect on perceived stress through each mediator, we will assess the outcome (perceived stress) and mediators (compassion, positive emotions, equanimity/mindfulness) for all participants at the midpoint of the six-week intervention (T2). This assessment schedule will allow us to more accurately determine the timing and order of change in each variable of interest. We will fit a parallel mediation model that includes group assignment (mindfulness vs. WLC) as assigned at T1 as the predictor variable, compassion, positive emotions, and equanimity/mindfulness from T2 as the mediators, and scores on the PSS at T3 as the outcome. The model will control for T1 perceived stress and T1 values of each mediator.

Based on recommendations by MacKinnon et al. (155), we will test the significance of the indirect effects using a non-parametric bootstrap approach (5,000 samples), which yields an average point estimate and a 95% confidence interval (CI) of the indirect, direct, and total effects. The effect is considered significant if the CI does not include zero (154).

## 11.3 Analyses for Specific Aims and Hypotheses

**Aim 1: Assess the feasibility of a mindfulness-based intervention based on the traditional Four Immeasurables practice for healthcare workers.**

*Hypothesis 1: We will be able to successfully recruit, enroll, and retain 80 full-time healthcare employees to participate in the trial. Participants randomized to the mindfulness intervention will be able to attend the majority (~75%) of the weekly group classes. Additionally, participants in the mindfulness intervention will find the course material relevant and beneficial.*

**Aim 2: Determine the effects of BEST vs. waitlist control on perceived stress.**

*Hypothesis 2: Those randomly assigned to participate in the mindfulness intervention will show a greater reduction in perceptions of stress (primary outcome) than those assigned to the WLC at the primary study endpoint (post-intervention; T3).*

Linear mixed models will be used to determine whether those randomized to the mindfulness condition (coded 1) experience greater reductions in perceptions of stress than those randomized to the WLC condition (coded 0). Models will include GROUP (mindfulness vs. WLC), TIME, a GROUP-by-TIME interaction, and demographic or occupational-related variables with baseline imbalances. To enhance interpretability, we will center all covariates (if necessary) at the grand mean before entering equations. The models will contain fixed effects for GROUP and TIME and random effects for individuals. The primary hypothesis is that participants in the mindfulness condition will show a greater reduction in perceived stress than participants in the WLC from baseline to post-intervention. Differences between conditions over time will be tested with the GROUP-by-TIME interaction. If statistically significant, the interaction term indicates that differences over time are a function of group assignment. We will probe significant GROUP-by-TIME interaction terms using the “pick-a-point” procedure outlined by Bauer and Curran (156) and Preacher et al. (157). We will calculate simple slopes for each group (mindfulness and WLC) at each time point to determine group differences at each value of the TIME variable (T1 and T3). Tests will be conducted at a significance level of 0.05.

**Aim 3: Evaluate the effects of BEST vs. waitlist control on negative and positive psychosocial symptoms experienced by healthcare workers.**

*Hypothesis 3: Those randomly assigned to participate in the mindfulness intervention will show greater reductions in depressive symptoms and burnout and greater improvements in psychological well-being and sleep quality than those assigned to the WLC.*

The same analytic approach used for Aim 2 will be applied here. Mixed models with GROUP, TIME, and a GROUP-by-TIME interaction term will examine changes in negative (depression and burnout) and positive (well-being and sleep quality) psychological characteristics as a function of group assignment. Tests will be conducted at a significance level of 0.05.

**Aim 4:** **Determine the effects of BEST vs. waitlist control on inflammatory processes associated with stress and relevant for long-term health.**

*Hypothesis 4: Those randomly assigned to participate in the mindfulness intervention will exhibit reduced inflammatory markers after the intervention relative to those assigned to the WLC.*

As with Aims 2 and 3, mixed models will be used to examine changes in markers of inflammatory activity (circulating protein markers and molecular pro-inflammatory gene expression and signaling) from pre- to post-intervention. Analyses will examine effects on individual markers of inflammation (e.g., IL-6) rather than combining multiple markers into an inflammatory composite. Effects on IL-6 and CRP are of particular interest, given previous investigations that have shown mindfulness intervention effects on these markers. As noted, these variables will likely need to be log-transformed to normalize distributions before analyses.

As previously noted, analyses will focus on changes from pre- to post-intervention in the expression of a set of 19 pro-inflammatory, 28 Type I interferon response, and three antibody synthesis genes in the BEST group compared to the WLC. A CTRA weighted score will be created, where inflammatory genes will be weighted +1 and antiviral and antibody-related genes will be weighted −1, as in previous research (125,158). CTRA-weighted scores are a continuous outcome and will be analyzed using the same mixed model approach described above.

As an additional measure of inflammatory activity, group differences in bioinformatically inferred NF-κB, AP-1, and CREB activity will be assessed using TELiS promoter-based bioinformatics analyses as used in previous studies (59,60,159). All transcripts showing greater than 1.5-fold change over time from pre- to post-intervention will serve as input into TELiS. For these analyses, the ratio of response element frequencies in the promoters of upregulated genes versus downregulated genes will be taken as a measure of the differential activity of transcription control pathways. Log ratios will be averaged over nine different parametric combinations of promoter length (-300, -600, and -1000 to +200 base pairs upstream of RefSeq-designated transcription start site) and motif detection stringency (TRANSFAC mat_sim values of .80, .90, and .95) to ensure robust results (160). The calculated log ratio is a continuous outcome that will be assessed using the same mixed model approach described above.

**Aim 5: Explore theoretically based mechanisms of the effect of the BEST intervention on the primary outcome, perceived stress (exploratory aim).**

*Hypothesis 5 (exploratory): Intervention effects on perceptions of stress will be mediated by compassion, positive emotions, and equanimity/mindfulness such that participants in the mindfulness intervention will experience increases in compassion, positive emotions, and equanimity/mindfulness, and those increases will mediate intervention effects on perceived stress.*

As an exploratory aim, a parallel mediation model will be fit, including group assignment (mindfulness vs. WLC) as assigned at T1 as the predictor variable, compassion, positive emotions, and equanimity/mindfulness from T2 as mediators, and scores on the PSS at T3 as the outcome. The model will control for T1 perceived stress and T1 values of each mediator.

The significance of the indirect effects will be tested using a non-parametric bootstrap approach (5,000 samples), which yields an average point estimate and a 95% CI of the indirect, direct, and total effects. The effect is presumed significant if the CI does not include zero (154).

# 12. REFERENCES

1.	Davis KG, Kotowski SE. Prevalence of Musculoskeletal Disorders for Nurses in Hospitals, Long-Term Care Facilities, and Home Health Care: A Comprehensive Review. Hum Factors J Hum Factors Ergon Soc. 2015 Aug;57(5):754–92.

2.	Garzaro G, Clari M, Ciocan C, Albanesi B, Guidetti G, Dimonte V, et al. Physical Health and Work Ability among Healthcare Workers. A Cross-Sectional Study. Nurs Rep. 2022 Apr 5;12(2):259–69.

3.	Pappa S, Ntella V, Giannakas T, Giannakoulis VG, Papoutsi E, Katsaounou P. Prevalence of depression, anxiety, and insomnia among healthcare workers during the COVID-19 pandemic: A systematic review and meta-analysis. Brain Behav Immun. 2020 Aug;88:901–7.

4.	Terlizzi E, Villarroel M. National Center for Health Statistics. 2020 [cited 2025 Jan 29]. Symptoms of generalized anxiety disorder among adults: United States, 2019. Available from: https://www.cdc.gov/nchs/products/databriefs/db378.htm

5.	Cao X, Chen L. Relationships between resilience, empathy, compassion fatigue, work engagement and turnover intention in haemodialysis nurses: A cross‐sectional study. J Nurs Manag. 2021 Jul;29(5):1054–63.

6.	Enns V, Currie S, Wang J. Professional autonomy and work setting as contributing factors to depression and absenteeism in Canadian nurses. Nurs Outlook. 2015 May;63(3):269–77.

7.	Han S, Shanafelt TD, Sinsky CA, Awad KM, Dyrbye LN, Fiscus LC, et al. Estimating the Attributable Cost of Physician Burnout in the United States. Ann Intern Med. 2019 Jun 4;170(11):784.

8.	Shanafelt TD, Dyrbye LN, West CP, Sinsky CA. Potential Impact of Burnout on the US Physician Workforce. Mayo Clin Proc. 2016 Nov;91(11):1667–8.

9.	Wells-English D, Giese J, Price J. Compassion Fatigue and Satisfaction: Influence on Turnover Among Oncology Nurses at an Urban Cancer Center. Clin J Oncol Nurs. 2019 Oct 1;23(5):487–93.

10.	Fahrenkopf AM, Sectish TC, Barger LK, Sharek PJ, Lewin D, Chiang VW, et al. Rates of medication errors among depressed and burnt out residents: prospective cohort study. BMJ. 2008 Mar 1;336(7642):488–91.

11.	Pereira-Lima K, Mata DA, Loureiro SR, Crippa JA, Bolsoni LM, Sen S. Association Between Physician Depressive Symptoms and Medical Errors: A Systematic Review and Meta-analysis. JAMA Netw Open. 2019 Nov 27;2(11):e1916097.

12.	Salyers MP, Bonfils KA, Luther L, Firmin RL, White DA, Adams EL, et al. The Relationship Between Professional Burnout and Quality and Safety in Healthcare: A Meta-Analysis. J Gen Intern Med. 2017 Apr;32(4):475–82.

13.	West CP, Huschka MM, Novotny PJ, Sloan JA, Kolars JC, Habermann TM, et al. Association of Perceived Medical Errors With Resident Distress and Empathy: A Prospective Longitudinal Study. JAMA. 2006 Sep 6;296(9):1071.

14.	Dewa CS, Loong D, Bonato S, Trojanowski L. The relationship between physician burnout and quality of healthcare in terms of safety and acceptability: a systematic review. BMJ Open. 2017 Jun;7(6):e015141.

15.	Halbesleben JRB, Rathert C. Linking physician burnout and patient outcomes: Exploring the dyadic relationship between physicians and patients. Health Care Manage Rev. 2008 Jan;33(1):29–39.

16.	Labrague LJ, De Los Santos JAA. Resilience as a mediator between compassion fatigue, nurses’ work outcomes, and quality of care during the COVID-19 pandemic. Appl Nurs Res. 2021 Oct;61:151476.

17.	Pérez‐García E, Ortega‐Galán ÁM, Ibáñez‐Masero O, Ramos‐Pichardo JD, Fernández‐Leyva A, Ruiz‐Fernández MD. Qualitative study on the causes and consequences of compassion fatigue from the perspective of nurses. Int J Ment Health Nurs. 2021 Apr;30(2):469–78.

18.	Awan S, Diwan MN, Aamir A, Allahuddin Z, Irfan M, Carano A, et al. Suicide in Healthcare Workers: Determinants, Challenges, and the Impact of COVID-19. Front Psychiatry. 2022 Feb 3;12:792925.

19.	Duarte D, El-Hagrassy MM, Couto TCE, Gurgel W, Fregni F, Correa H. Male and Female Physician Suicidality: A Systematic Review and Meta-analysis. JAMA Psychiatry. 2020 Jun 1;77(6):587.

20.	Schernhammer ES, Colditz GA. Suicide Rates Among Physicians: A Quantitative and Gender Assessment (Meta-Analysis). Am J Psychiatry. 2004 Dec 1;161(12):2295–302.

21.	Oreskovich MR. Prevalence of Alcohol Use Disorders Among American Surgeons. Arch Surg. 2012 Feb 1;147(2):168.

22.	Austin W, Goble E, Leier B, Byrne P. Compassion Fatigue: The Experience of Nurses. Ethics Soc Welf. 2009 Jul;3(2):195–214.

23.	Nolte AG, Downing C, Temane A, Hastings‐Tolsma M. Compassion fatigue in nurses: A metasynthesis. J Clin Nurs. 2017 Dec;26(23–24):4364–78.

24.	Steinheiser M. Compassion fatigue among nurses in skilled nursing facilities: Discoveries and challenges of a conceptual model in research. Appl Nurs Res. 2018 Dec;44:97–9.

25.	Adams A, Alhujaily E, Anil V, Kandah O, Patrick-Eisenberg A, Decker K, et al. Healthcare Provider Burnout: Red Flag for a Rising Epidemic. South Med J. 2024 Nov;117(11):629–33.

26.	De Hert S. Burnout in Healthcare Workers: Prevalence, Impact and Preventative Strategies. Local Reg Anesth. 2020 Oct;Volume 13:171–83.

27.	Fond G, Fernandes S, Lucas G, Greenberg N, Boyer L. Depression in healthcare workers: Results from the nationwide AMADEUS survey. Int J Nurs Stud. 2022 Nov;135:104328.

28.	Rotenstein LS, Torre M, Ramos MA, Rosales RC, Guille C, Sen S, et al. Prevalence of Burnout Among Physicians: A Systematic Review. JAMA. 2018 Sep 18;320(11):1131.

29.	West CP. Association of Resident Fatigue and Distress With Perceived Medical Errors. JAMA. 2009 Sep 23;302(12):1294.

30.	Hojat M, Louis DZ, Markham FW, Wender R, Rabinowitz C, Gonnella JS. Physiciansʼ Empathy and Clinical Outcomes for Diabetic Patients: Acad Med. 2011 Mar;86(3):359–64.

31.	Lee HF, Kuo CC, Chien TW, Wang YR. A Meta-Analysis of the Effects of Coping Strategies on Reducing Nurse Burnout. Appl Nurs Res. 2016 Aug;31:100–10.

32.	Patole S, Pawale D, Rath C. Interventions for Compassion Fatigue in Healthcare Providers—A Systematic Review of Randomised Controlled Trials. Healthcare. 2024 Jan 11;12(2):171.

33.	West CP, Dyrbye LN, Erwin PJ, Shanafelt TD. Interventions to prevent and reduce physician burnout: a systematic review and meta-analysis. The Lancet. 2016 Nov;388(10057):2272–81.

34.	Zaçe D, Hoxhaj I, Orfino A, Viteritti AM, Janiri L, Di Pietro ML. Interventions to address mental health issues in healthcare workers during infectious disease outbreaks: A systematic review. J Psychiatr Res. 2021 Apr;136:319–33.

35.	Kabat-Zinn J. Wherever you go, there you are: mindfulness meditation in everyday life. New York: Hyperion; 2005. 280 p.

36.	Segal ZV, Williams JMG, Teasdale JD. Mindfulness-based cognitive therapy for depression: a new approach to preventing relapse. New York: Guilford Press; 2002. 351 p.

37.	Buddhaghoṣa, Ñāṇamoli. The Path of Purification: the classic manual of Buddhist doctrine and meditation. Onalaska: BPS Pariyatti Editions; 2000.

38.	Wallace BA, Houshmand Z. The four immeasurables: practices to open the heart. Third edition. Ithaca, New York: Snow Lion Publications; 2010. 186 p.

39.	Fredrickson BL. The role of positive emotions in positive psychology: The broaden-and-build theory of positive emotions. Am Psychol. 2001 Mar;56(3):218–26.

40.	Galante J, Galante I, Bekkers MJ, Gallacher J. Effect of kindness-based meditation on health and well-being: A systematic review and meta-analysis. J Consult Clin Psychol. 2014 Dec;82(6):1101–14.

41.	Hofmann SG, Grossman P, Hinton DE. Loving-kindness and compassion meditation: Potential for psychological interventions. Clin Psychol Rev. 2011 Nov 1;31(7):1126–32.

42.	Zeng X, Wang R, Oei TPS, Leung FYK. Heart of Joy: a Randomized Controlled Trial Evaluating the Effect of an Appreciative Joy Meditation Training on Subjective Well-Being and Attitudes. Mindfulness. 2019 Mar;10(3):506–15.

43.	Pace TWW, Negi LT, Adame DD, Cole SP, Sivilli TI, Brown TD, et al. Effect of compassion meditation on neuroendocrine, innate immune and behavioral responses to psychosocial stress. Psychoneuroendocrinology. 2009 Jan;34(1):87–98.

44.	Pace TWW, Negi LT, Sivilli TI, Issa MJ, Cole SP, Adame DD, et al. Innate immune, neuroendocrine and behavioral responses to psychosocial stress do not predict subsequent compassion meditation practice time. Psychoneuroendocrinology. 2010 Feb;35(2):310–5.

45.	Le Nguyen KD, Lin J, Algoe SB, Brantley MM, Kim SL, Brantley J, et al. Loving-kindness meditation slows biological aging in novices: Evidence from a 12-week randomized controlled trial. Psychoneuroendocrinology. 2019 Oct;108:20–7.

46.	Black PH, Garbutt LD. Stress, inflammation and cardiovascular disease. J Psychosom Res. 2002 Jan;52(1):1–23.

47.	Segerstrom SC, Miller GE. Psychological Stress and the Human Immune System: A Meta-Analytic Study of 30 Years of Inquiry. Psychol Bull. 2004;130(4):601–30.

48.	Wellen KE, Hotamisligil GS. Inflammation, stress, and diabetes. J Clin Invest. 2005 May 2;115(5):1111–9.

49.	Eisenberger NI, Moieni M, Inagaki TK, Muscatell KA, Irwin MR. In Sickness and in Health: The Co-Regulation of Inflammation and Social Behavior. Neuropsychopharmacology. 2017 Jan;42(1):242–53.

50.	Mekhora C, Lamport DJ, Spencer JPE. An overview of the relationship between inflammation and cognitive function in humans, molecular pathways and the impact of nutraceuticals. Neurochem Int. 2024 Dec;181:105900.

51.	Moriarity DP. A primer on common analytic concerns in psychoneuroimmunology: Alternatives and paths forward. Brain Behav Immun. 2022 May;102:338–40.

52.	Creswell JD, Lindsay EK. How Does Mindfulness Training Affect Health? A Mindfulness Stress Buffering Account. Curr Dir Psychol Sci. 2014 Dec;23(6):401–7.

53.	Gu J, Strauss C, Bond R, Cavanagh K. How do mindfulness-based cognitive therapy and mindfulness-based stress reduction improve mental health and wellbeing? A systematic review and meta-analysis of mediation studies. Clin Psychol Rev. 2015 Apr;37:1–12.

54.	Hölzel BK, Lazar SW, Gard T, Schuman-Olivier Z, Vago DR, Ott U. How Does Mindfulness Meditation Work? Proposing Mechanisms of Action From a Conceptual and Neural Perspective. Perspect Psychol Sci. 2011 Nov;6(6):537–59.

55.	Lindsay EK, Creswell JD. Mechanisms of mindfulness training: Monitor and Acceptance Theory (MAT). Clin Psychol Rev. 2017 Feb;51:48–59.

56.	Lindsay EK, Young S, Creswell JD. Mindfulness training fosters a positive outlook during acute stress: A randomized controlled trial. Emotion [Internet]. 2024 Nov 21 [cited 2025 May 22]; Available from: https://doi.apa.org/doi/10.1037/emo0001452

57.	Scafuto F, Quinto RM, Ghiroldi S, Montecucco NF, Presaghi F, Iani L, et al. The mediation role of emotion regulation strategies on the relationship between mindfulness effects, psychological well-being and distress among youths: findings from a randomized controlled trial. Curr Psychol. 2024 Aug;43(29):24295–307.

58.	Teper R, Inzlicht M. Meditation, mindfulness and executive control: the importance of emotional acceptance and brain-based performance monitoring. Soc Cogn Affect Neurosci. 2013 Jan 1;8(1):85–92.

59.	Bower JE, Crosswell AD, Stanton AL, Crespi CM, Winston D, Arevalo J, et al. Mindfulness meditation for younger breast cancer survivors: A randomized controlled trial: Mindfulness for Young Cancer Survivors. Cancer. 2015 Apr 15;121(8):1231–40.

60.	Creswell JD, Irwin MR, Burklund LJ, Lieberman MD, Arevalo JMG, Ma J, et al. Mindfulness-Based Stress Reduction training reduces loneliness and pro-inflammatory gene expression in older adults: A small randomized controlled trial. Brain Behav Immun. 2012 Oct;26(7):1095–101.

61.	Creswell JD, Taren AA, Lindsay EK, Greco CM, Gianaros PJ, Fairgrieve A, et al. Alterations in Resting-State Functional Connectivity Link Mindfulness Meditation With Reduced Interleukin-6: A Randomized Controlled Trial. Biol Psychiatry. 2016 Jul;80(1):53–61.

62.	Montero-Marin J, Andrés-Rodríguez L, Tops M, Luciano JV, Navarro-Gil M, Feliu-Soler A, et al. Effects of attachment-based compassion therapy (ABCT) on brain-derived neurotrophic factor and low-grade inflammation among fibromyalgia patients: A randomized controlled trial. Sci Rep [Internet]. 2019 Oct 30 [cited 2025 May 22];9(1). Available from: https://www.nature.com/articles/s41598-019-52260-z

63.	Dunn TJ, Dimolareva M. The effect of mindfulness-based interventions on immunity-related biomarkers: a comprehensive meta-analysis of randomised controlled trials. Clin Psychol Rev. 2022 Mar;92:102124.

64.	Dimidjian S, Segal ZV. Prospects for a clinical science of mindfulness-based intervention. Am Psychol. 2015 Oct;70(7):593–620.

65.	Britton WB, Desbordes G, Acabchuk R, Peters S, Lindahl JR, Canby NK, et al. From Self-Esteem to Selflessness: An Evidence (Gap) Map of Self-Related Processes as Mechanisms of Mindfulness-Based Interventions. Front Psychol. 2021 Nov 22;12:730972.

66.	Goldberg SB. A common factors perspective on mindfulness-based interventions. Nat Rev Psychol. 2022 Aug 1;1(10):605–19.

67.	Dalai Lama. The power of compassion: a collection of lectures by His Holiness the XIV Dalai Lama. 6. [Aufl.]. London: Thorsons; 1995. 215 p.

68.	Bränström R, Duncan LG, Moskowitz JT. The association between dispositional mindfulness, psychological well-being, and perceived health in a Swedish population-based sample: Influence of dispositional mindfulness. Br J Health Psychol. 2011 May;16(2):300–16.

69.	Krieger T, Berger T, Holtforth MG. The relationship of self-compassion and depression: Cross-lagged panel analyses in depressed patients after outpatient therapy. J Affect Disord. 2016 Sep;202:39–45.

70.	López A, Sanderman R, Schroevers MJ. A Close Examination of the Relationship Between Self-Compassion and Depressive Symptoms. Mindfulness. 2018 Oct;9(5):1470–8.

71.	Morris BH, Bylsma LM, Rottenberg J. Does emotion predict the course of major depressive disorder? A review of prospective studies. Br J Clin Psychol. 2009 Sep;48(3):255–73.

72.	Saarinen A, Keltikangas-Järvinen L, Cloninger CR, Veijola J, Elovainio M, Lehtimäki T, et al. The relationship of dispositional compassion for others with depressive symptoms over a 15-year prospective follow-up. J Affect Disord. 2019 May;250:354–62.

73.	Vanderlind WM, Millgram Y, Baskin-Sommers AR, Clark MS, Joormann J. Understanding positive emotion deficits in depression: From emotion preferences to emotion regulation. Clin Psychol Rev. 2020 Mar;76:101826.

74.	Williams JMG. Mindfulness, Depression and Modes of Mind. Cogn Ther Res. 2008 Dec;32(6):721–33.

75.	Benedek DM, Fullerton C, Ursano RJ. First Responders: Mental Health Consequences of Natural and Human-Made Disasters for Public Health and Public Safety Workers. Annu Rev Public Health. 2007 Apr 1;28(1):55–68.

76.	Tan L, Petrie K, Deady M, Bryant RA, Harvey SB. Systematic review of first responder post-deployment or post-incident psychosocial interventions. Occup Med. 2022 Apr 19;72(3):160–9.

77.	Goldberg SB, Tucker RP, Greene PA, Davidson RJ, Wampold BE, Kearney DJ, et al. Mindfulness-based interventions for psychiatric disorders: A systematic review and meta-analysis. Clin Psychol Rev. 2018 Feb;59:52–60.

78.	Goldberg SB, Riordan KM, Sun S, Davidson RJ. The Empirical Status of Mindfulness-Based Interventions: A Systematic Review of 44 Meta-Analyses of Randomized Controlled Trials. Perspect Psychol Sci. 2022 Jan;17(1):108–30.

79.	Querstret D, Morison L, Dickinson S, Cropley M, John M. Mindfulness-based stress reduction and mindfulness-based cognitive therapy for psychological health and well-being in nonclinical samples: A systematic review and meta-analysis. Int J Stress Manag. 2020 Nov;27(4):394–411.

80.	Chen C, Meier ST. Burnout and depression in nurses: A systematic review and meta-analysis. Int J Nurs Stud. 2021 Dec;124:104099.

81.	Hofmann SG, Gómez AF. Mindfulness-Based Interventions for Anxiety and Depression. Psychiatr Clin North Am. 2017 Dec;40(4):739–49.

82.	Nagarajan R, Ramachandran P, Dilipkumar R, Kaur P. Global estimate of burnout among the public health workforce: a systematic review and meta-analysis. Hum Resour Health. 2024 May 21;22(1):30.

83.	Bamforth K, Rae P, Maben J, Lloyd H, Pearce S. Perceptions of healthcare professionals’ psychological wellbeing at work and the link to patients’ experiences of care: A scoping review. Int J Nurs Stud Adv. 2023 Dec;5:100148.

84.	Hall LH, Johnson J, Watt I, Tsipa A, O’Connor DB. Healthcare Staff Wellbeing, Burnout, and Patient Safety: A Systematic Review. Harris F, editor. PLOS ONE. 2016 Jul 8;11(7):e0159015.

85.	Janatolmakan M, Naghipour A, Khatony A. Prevalence and factors associated with poor sleep quality among nurses in COVID-19 wards. Sci Rep. 2024 Jul 18;14(1):16616.

86.	Qiu D, Yu Y, Li RQ, Li YL, Xiao SY. Prevalence of sleep disturbances in Chinese healthcare professionals: a systematic review and meta-analysis. Sleep Med. 2020 Mar;67:258–66.

87.	Zeng LN, Yang Y, Wang C, Li XH, Xiang YF, Hall BJ, et al. Prevalence of Poor Sleep Quality in Nursing Staff: A Meta-Analysis of Observational Studies. Behav Sleep Med. 2020 Nov 1;18(6):746–59.

88.	Franzen PL, Buysse DJ. Sleep disturbances and depression: risk relationships for subsequent depression and therapeutic implications. Dialogues Clin Neurosci. 2008 Dec 31;10(4):473–81.

89.	Nutt D, Wilson S, Paterson L. Sleep disorders as core symptoms of depression. Dialogues Clin Neurosci. 2008 Sep 30;10(3):329–36.

90.	Garland SN, Britton W, Agagianian N, Goldman RE, Carlson LE, Ong JC. Mindfulness, Affect, and Sleep. In: Sleep and Affect [Internet]. Elsevier; 2015 [cited 2023 Mar 2]. p. 339–73. Available from: https://linkinghub.elsevier.com/retrieve/pii/B9780124171886000165

91.	Garland SN, Zhou ES, Gonzalez BD, Rodriguez N. The Quest for Mindful Sleep: a Critical Synthesis of the Impact of Mindfulness-Based Interventions for Insomnia. Curr Sleep Med Rep. 2016 Sep;2(3):142–51.

92.	Cohen S, Kamarck T, Mermelstein R. A Global Measure of Perceived Stress. J Health Soc Behav. 1983 Dec;24(4):385.

93.	Qiu F, Liang CL, Liu H, Zeng YQ, Hou S, Huang S, et al. Impacts of cigarette smoking on immune responsiveness: Up and down or upside down? Oncotarget. 2017 Jan 3;8(1):268–84.

94.	Smalley SL, Winston D. Fully present: the science, art, and practice of mindfulness. First Hachette Go edition. New York: Hachette Go; 2022.

95.	Bellg AJ, Treatment Fidelity Workgroup of the NIH Behavior Change Consortium, Borrelli B, Resnick B, Hecht J, Minicucci DS, et al. Enhancing Treatment Fidelity in Health Behavior Change Studies: Best Practices and Recommendations From the NIH Behavior Change Consortium. Health Psychol. 2004;23(5):443–51.

96.	Cox BJ, Fergus KD, Swinson RP. Patient satisfaction with behavioral treatments for panic disorder with agoraphobia. J Anxiety Disord. 1994 Jul;8(3):193–206.

97.	Babore A, Lombardi L, Viceconti ML, Pignataro S, Marino V, Crudele M, et al. Psychological effects of the COVID-2019 pandemic: Perceived stress and coping strategies among healthcare professionals. Psychiatry Res. 2020 Nov;293:113366.

98.	Karabulut N, Gürçayır D, Yaman Aktaş Y, Kara A, Kızıloğlu B, Arslan B, et al. The effect of perceived stress on anxiety and sleep quality among healthcare professionals in intensive care units during the coronavirus pandemic. Psychol Health Med. 2021 Jan 2;26(1):119–30.

99.	Nyklíček I, Kuijpers KF. Effects of Mindfulness-Based Stress Reduction Intervention on Psychological Well-being and Quality of Life: Is Increased Mindfulness Indeed the Mechanism? Ann Behav Med. 2008 Jun;35(3):331–40.

100.	Radloff LS. The CES-D Scale: A Self-Report Depression Scale for Research in the General Population. Appl Psychol Meas. 1977 Jun;1(3):385–401.

101.	Goebert D, Thompson D, Takeshita J, Beach C, Bryson P, Ephgrave K, et al. Depressive Symptoms in Medical Students and Residents: A Multischool Study: Acad Med. 2009 Feb;84(2):236–41.

102.	Bower JE, Partridge AH, Wolff AC, Thorner ED, Irwin MR, Joffe H, et al. Targeting Depressive Symptoms in Younger Breast Cancer Survivors: The Pathways to Wellness Randomized Controlled Trial of Mindfulness Meditation and Survivorship Education. J Clin Oncol. 2021 Nov 1;39(31):3473–84.

103.	Maslach C, Jackson S, Leiter M. The Maslach Burnout Inventory Manual. In: Evaluating Stress: A Book of Resources. 1997. p. 191–218.

104.	Hallberg UE, Sverke M. Construct Validity of the Maslach Burnout Inventory: Two Swedish Health Care Samples. Eur J Psychol Assess. 2004 Jan;20(4):320–38.

105.	Kalliath TJ, O’Driscoll MP, Gillespie DF, Bluedorn AC. A test of the Maslach Burnout Inventory in three samples of healthcare professionals. Work Stress. 2000 Jan;14(1):35–50.

106.	Brady KJS, Ni P, Sheldrick RC, Trockel MT, Shanafelt TD, Rowe SG, et al. Describing the emotional exhaustion, depersonalization, and low personal accomplishment symptoms associated with Maslach Burnout Inventory subscale scores in US physicians: an item response theory analysis. J Patient-Rep Outcomes. 2020 Dec;4(1):42.

107.	Poghosyan L, Aiken LH, Sloane DM. Factor structure of the Maslach burnout inventory: An analysis of data from large scale cross-sectional surveys of nurses from eight countries. Int J Nurs Stud. 2009 Jul;46(7):894–902.

108.	Musker M, Othman S. Effective interventions to reduce burnout in nurses: A meta-analysis. Complement Ther Clin Pract. 2024 Feb;54:101827.

109.	Keyes CLM. The Mental Health Continuum: From Languishing to Flourishing in Life. J Health Soc Behav. 2002 Jun;43(2):207.

110.	Lamers SMA, Westerhof GJ, Bohlmeijer ET, Ten Klooster PM, Keyes CLM. Evaluating the psychometric properties of the mental health Continuum‐Short Form (MHC‐SF). J Clin Psychol. 2011 Jan;67(1):99–110.

111.	Keyes CLM, Shapiro AD. How Healthy Are We? In: Brim OG, Ryff CD, Kessler RC, editors. A National Study of Well-Being at Midlife [Internet]. University of Chicago Press; 2003 [cited 2025 Feb 20]. p. 350–72. Available from: https://doi.org/10.7208/9780226074764-013

112.	Franken K, Lamers SMA, Ten Klooster PM, Bohlmeijer ET, Westerhof GJ. Validation of the Mental Health Continuum-Short Form and the dual continua model of well-being and psychopathology in an adult mental health setting. J Clin Psychol. 2018 Dec;74(12):2187–202.

113.	Kraiss J, Redelinghuys K, Weiss LauraA. The effects of psychological interventions on well-being measured with the Mental Health Continuum: a meta-analysis. J Happiness Stud. 2022 Oct;23(7):3655–89.

114.	Morin CM, Belleville G, Bélanger L, Ivers H. The Insomnia Severity Index: Psychometric Indicators to Detect Insomnia Cases and Evaluate Treatment Response. Sleep. 2011 May;34(5):601–8.

115.	Aydin Guclu O, Karadag M, Akkoyunlu ME, Acican T, Sertogullarindan B, Kirbas G, et al. Association between burnout, anxiety and insomnia in healthcare workers: a cross-sectional study. Psychol Health Med. 2022 May 28;27(5):1117–30.

116.	Ong JC, Manber R, Segal Z, Xia Y, Shapiro S, Wyatt JK. A Randomized Controlled Trial of Mindfulness Meditation for Chronic Insomnia. Sleep. 2014 Sep 1;37(9):1553–63.

117.	Black DS, Slavich GM. Mindfulness meditation and the immune system: a systematic review of randomized controlled trials: Mindfulness meditation and the immune system. Ann N Y Acad Sci. 2016 Jun;1373(1):13–24.

118.	Bower JE, Irwin MR. Mind–body therapies and control of inflammatory biology: A descriptive review. Brain Behav Immun. 2016 Jan;51:1–11.

119.	González-Moret R, Cebolla A, Cortés X, Baños RM, Navarrete J, De La Rubia JE, et al. The effect of a mindfulness-based therapy on different biomarkers among patients with inflammatory bowel disease: a randomised controlled trial. Sci Rep. 2020 Apr 8;10(1):6071.

120.	Villalba DK, Lindsay EK, Marsland AL, Greco CM, Young S, Brown KW, et al. Mindfulness training and systemic low-grade inflammation in stressed community adults: Evidence from two randomized controlled trials. PloS One. 2019;14(7):e0219120.

121.	Walsh E, Eisenlohr-Moul T, Baer R. Brief mindfulness training reduces salivary IL-6 and TNF-α in young women with depressive symptomatology. J Consult Clin Psychol. 2016 Oct;84(10):887–97.

122.	Meso Scale Diagnostics, LLC. Meso Scale Discovery multiplex immunoassay [Internet]. 2023. Available from: https://www.mesoscale.com/en/products_and_services/assay_kits/multiplex_assay_kits

123.	R&D Systems. R&D Systems® ELISA Kits [Internet]. [cited 2023 Mar 17]. Available from: https://www.rndsystems.com/products/elisas

124.	Cole SW. The Conserved Transcriptional Response to Adversity. Curr Opin Behav Sci. 2019 Aug;28:31–7.

125.	Boyle CC, Cole SW, Dutcher JM, Eisenberger NI, Bower JE. Changes in eudaimonic well-being and the conserved transcriptional response to adversity in younger breast cancer survivors. Psychoneuroendocrinology. 2019 May;103:173–9.

126.	Walton KG, Wenuganen S, Cole SW. Transcendental Meditation practitioners show reduced expression of the Conserved Transcriptional Response to Adversity. Brain Behav Immun - Health. 2023 Oct;32:100672.

127.	Cole SW, Hawkley LC, Arevalo JMG, Cacioppo JT. Transcript origin analysis identifies antigen-presenting cells as primary targets of socially regulated gene expression in leukocytes. Proc Natl Acad Sci. 2011 Feb 15;108(7):3080–5.

128.	Raes F, Pommier E, Neff KD, Van Gucht D. Construction and factorial validation of a short form of the Self‐Compassion Scale. Clin Psychol Psychother. 2011 May;18(3):250–5.

129.	Pommier E, Neff KD, Tóth-Király I. The Development and Validation of the Compassion Scale. Assessment. 2020 Jan;27(1):21–39.

130.	Neff K. Self-Compassion: An Alternative Conceptualization of a Healthy Attitude Toward Oneself. Self Identity. 2003 Apr;2(2):85–101.

131.	Neff KD, Knox MC, Long P, Gregory K. Caring for others without losing yourself: An adaptation of the Mindful Self‐Compassion Program for Healthcare Communities. J Clin Psychol. 2020 Sep;76(9):1543–62.

132.	Demirel Y, Çağatay HT, Ertuğrul B, Başaran E, Salimoğlu S. The effect of the compassion levels of healthcare workers on their psychological well-being. Work. 2024 Jan 12;77(1):253–62.

133.	Fredrickson BL. Positive Emotions Broaden and Build. In: Advances in Experimental Social Psychology [Internet]. Elsevier; 2013 [cited 2024 Aug 22]. p. 1–53. Available from: https://linkinghub.elsevier.com/retrieve/pii/B9780124072367000012

134.	Fredrickson BL. What Good Are Positive Emotions? Rev Gen Psychol. 1998 Sep;2(3):300–19.

135.	Rink LC, Silva SG, Adair KC, Oyesanya TO, Humphreys JC, Sexton JB. Characterizing burnout and resilience among nurses: A latent profile analysis of emotional exhaustion, emotional thriving and emotional recovery. Nurs Open. 2023 Nov;10(11):7279–91.

136.	Fredrickson BL, Cohn MA, Coffey KA, Pek J, Finkel SM. Open hearts build lives: Positive emotions, induced through loving-kindness meditation, build consequential personal resources. J Pers Soc Psychol. 2008 Nov;95(5):1045–62.

137.	Fredrickson BL, Boulton AJ, Firestine AM, Van Cappellen P, Algoe SB, Brantley MM, et al. Positive Emotion Correlates of Meditation Practice: a Comparison of Mindfulness Meditation and Loving-Kindness Meditation. Mindfulness. 2017 Dec;8(6):1623–33.

138.	Rogers HT, Shires AG, Cayoun BA. Development and Validation of the Equanimity Scale-16. Mindfulness. 2021 Jan;12(1):107–20.

139.	Shires A, Osborne S, Cayoun BA, Williams E, Rogers K. Predictive Validity and Response Shift in the Equanimity Scale-16. Mindfulness. 2023 Dec;14(12):2880–93.

140.	Baer RA, Smith GT, Hopkins J, Krietemeyer J, Toney L. Using Self-Report Assessment Methods to Explore Facets of Mindfulness. Assessment. 2006 Mar;13(1):27–45.

141.	Baer RA, Smith GT, Lykins E, Button D, Krietemeyer J, Sauer S, et al. Construct Validity of the Five Facet Mindfulness Questionnaire in Meditating and Non-Meditating Samples. Assessment. 2008 Sep;15(3):329–42.

142.	Arthur D, Dizon D, Jooste K, Li Z, Salvador M, Yao X. Mindfulness in nursing students: The five facet mindfulness questionnaire in samples of nursing students in China, the Philippines, and South Africa. Int J Ment Health Nurs. 2018 Jun;27(3):975–86.

143.	Kaisti I, Kulmala P, Hintsanen M, Hurtig T, Repo S, Paunio T, et al. The effects of mindfulness-based interventions in medical students: a systematic review. Adv Health Sci Educ. 2024 Mar;29(1):245–71.

144.	Aikens KA, Astin J, Pelletier KR, Levanovich K, Baase CM, Park YY, et al. Mindfulness Goes to Work: Impact of an Online Workplace Intervention. J Occup Environ Med. 2014 Jul;56(7):721–31.

145.	Faul F, Erdfelder E, Buchner A, Lang AG. Statistical power analyses using G*Power 3.1: Tests for correlation and regression analyses. Behav Res Methods. 2009 Nov;41(4):1149–60.

146.	Lam SU, Kirvin-Quamme A, Goldberg SB. Overall and Differential Attrition in Mindfulness-Based Interventions: a Meta-Analysis. Mindfulness. 2022 Nov;13(11):2676–90.

147.	Golden HL, Vosper J, Kingston J, Ellett L. The Impact of Mindfulness-Based Programmes on Self-Compassion in Nonclinical Populations: a Systematic Review and Meta-Analysis. Mindfulness. 2021 Jan;12(1):29–52.

148.	Petrovic J, Mettler J, Cho S, Heath NL. The effects of loving-kindness interventions on positive and negative mental health outcomes: A systematic review and meta-analysis. Clin Psychol Rev. 2024 Jun;110:102433.

149.	Wasson RS, Barratt C, O’Brien WH. Effects of Mindfulness-Based Interventions on Self-compassion in Health Care Professionals: a Meta-analysis. Mindfulness. 2020 Aug;11(8):1914–34.

150.	Gupta S. Intention-to-treat concept: A review. Perspect Clin Res. 2011;2(3):109.

151.	Grund, S., Lüdtke, O., & Robitzsch, A. Missing data in multilevel research. In: Humphrey SE, LeBreton JM, editors. The handbook of multilevel theory, measurement, and analysis [Internet]. Washington: American Psychological Association; 2019 [cited 2025 Feb 24]. p. 365–86. Available from: https://content.apa.org/books/16093-017

152.	Newman DA. Missing Data: Five Practical Guidelines. Organ Res Methods. 2014 Oct;17(4):372–411.

153.	R Core Team. R: A language and environment for statistical computing [Internet]. Vienna, Austria: R Foundation for Statistical Computing; 2022. Available from: https://www.R-project.org/

154.	Hayes AF. Introduction to mediation, moderation, and conditional process analysis: a regression-based approach. Third edition. New York, NY: The Guilford Press; 2022. (Methodology in the social sciences).

155.	MacKinnon DP, Lockwood CM, Williams J. Confidence Limits for the Indirect Effect: Distribution of the Product and Resampling Methods. Multivar Behav Res. 2004 Jan;39(1):99–128.

156.	Bauer DJ, Curran PJ. Probing Interactions in Fixed and Multilevel Regression: Inferential and Graphical Techniques. Multivar Behav Res. 2005 Jul;40(3):373–400.

157.	Preacher KJ, Curran PJ, Bauer DJ. Computational Tools for Probing Interactions in Multiple Linear Regression, Multilevel Modeling, and Latent Curve Analysis. J Educ Behav Stat. 2006 Dec;31(4):437–48.

158.	Cole SW, Levine ME, Arevalo JMG, Ma J, Weir DR, Crimmins EM. Loneliness, eudaimonia, and the human conserved transcriptional response to adversity. Psychoneuroendocrinology. 2015 Dec;62:11–7.

159.	Vandenbogaart E, Figueroa M, Winston D, Cole S, Bower J, Hsu JJ. Preliminary evaluation of a mindfulness intervention program in women with long COVID dysautonomia symptoms. Brain Behav Immun - Health. 2025 Mar;44:100963.

160.	Cole SW, Yan W, Galic Z, Arevalo J, Zack JA. Expression-based monitoring of transcription factor activity: the TELiS database. Bioinformatics. 2005 Mar 15;21(6):803–10.

# 12. APPENDIX A

*Study Flow Diagram*

# 13. APPENDIX B

*ANOVA: Fixed effects, main effects, and interaction term:*

*MANOVA: Repeated measures, within-between interaction term:*

*MANOVA: Repeated measures, between factors only:*

*ANOVA: Repeated measures, between factors only:*
